# Supplementary material for: Taking charge of eczema self-management: a qualitative interview study with young people with eczema
Source: BMJ Open. 2021 Jan 6;11(1):e044005. doi: 10.1136/bmjopen-2020-044005 (PMC7789449; doi:10.1136/bmjopen-2020-044005)
Supplement: Supplementary data [file bmjopen-2020-044005supp001.pdf]

Supplementary File 1 - Interview Schedule for SKINS Project

Part 1 of the interview: open ended/free-flow

- Could you tell me about your experiences of having [skin condition(s)]. You can say as a little or as much as you like about this.

Part 2 of the interview: follow-up and prompts

Realising that you had skin condition(s)

- What it was like when you first noticed that you might have a skin-related condition (or multiple skin-related conditions)?
- How long after you noticing/having symptoms did you talk to someone else about it?
- Have you been to see a doctor about your [skin condition(s)]? If so, at what stage did you first go?

Early knowledge/information about skin condition(s)

- What was it like when you were told you had [skin condition(s)]?
- Do you recall what, if anything, you knew about [skin condition(s)] before your diagnosis?
- After being diagnosed, did you look for other/more information elsewhere? If so, could you tell me about it?

Medical encounters/meetings

- Could I ask a bit more about the healthcare you've received for your [skin condition(s)]? So things like who do you see, where, how often?
- *If relevant:* What has it been like to move from a paediatric clinic to teenager and/or onto an adult clinic?

Greenwell K, *et al. BMJ Open* 2021; 11:e044005. doi: 10.1136/bmjopen-2020-044005

- Could you tell me about what happens when you go see [various health professionals]?

Treatments and managements of skin condition(s)

- Could you tell me about the treatments you have had in the past and any that you now do please?
- What were these treatments like? What did they involve?
- Did you have to make any decisions about these treatments? Like choosing between different options?
- Does anyone help you do these treatments? Or provide instructions about what you needed to do? Do they provide other kinds of practical help around your treatment?
- Are there some treatments or things to manage your [skin condition(s)] which are easier to do than others?
- Are there any which are particularly difficult or upsetting to do?
- Have there been any physical side effects to the treatments for your [skin condition(s)]?
- Have the treatments had any impact on the kinds of activities you can or would like to do?
- Have the treatments you've used had any other changes or prompted concerns for you?

Changes to the skin condition over time and in the anticipated future

- Could you run me through a typical day/week/month from the point of view of managing [skin condition(s)]?
- Have there been any changes over the time you've had [skin condition(s)] as to what the condition has been like (better, worse)? Either in relation to treatments or unexpectedly.

- Are there any more treatments planned or possible options for management in the future for you?

Everyday life as a young person with [skin condition(s)]

- Could you tell me what it is like being a young person with [skin condition(s)] (as opposed to a child or older adult with this)?
- *If relevant:* Do you think that [skin condition(s)] has an impact for you in terms of school/ college/university?
- *If relevant:* How about any impact in relation to employers (full-time, part-time)? This could be present, past and future/prospective employers.
- Are there any ways in which [skin condition(s)] plays a part in your friendships? This might include friendships at school/college/uni and/or at work but also beyond (including online)?
- Could I ask about any impacts of [skin condition(s)] on (potential and/or actual) romantic relationships?
- In your experience, does having [skin condition(s)] make a difference with regards to social situations or events you might attend? In a negative way or in a positive way?
- Are there any particular places you might go for which are affected in any way by [skin condition(s)]? For example, any places/spaces in particular that you would avoid? Or places where you feel more comfortable? Places you've gone to because of [skin condition(s)]?

Home life and [skin condition(s)]

- Are there any other aspects of your home life and lifestyle that you think having [skin condition(s)] affects? If so, how and why?
- What about any impact on your family [parents, siblings etc.] and/or those you live with [e.g. if in independent accommodation]?

‘Body work’ and body-image

- Does your [skin condition(s)] affect the things you can wear or otherwise put on your body/skin? For example cosmetics, make-up, clothes, piercings, tattoos, hair dying, depilation.

Subsequent information and support

- Could you tell me about any practical and/or emotional support you’ve found or received, either from individuals, organisations or websites?
- Are you aware of any support groups either for your condition or more generally for young people?

Sharing information/messages to others

- What would be your advice to other young people with [skin condition(s)]? For example, is there anything from your experience that you think they might be interested to know or might find helpful?
- Is there anything you wish you had known, or anything you still don’t know and would like to, about [skin condition(s)]? Are there things you’d like to know about others experiences?
- Based on your experience of [skin condition(s)], is there anything you think doctors and nurses should know? What would be your message to them?

Closing

- Is there anything else about your experience of [skin condition(s)] that I haven’t covered but that you think might be important? Or would you like to add to something you’ve said previously?

Greenwell K, *et al. BMJ Open* 2021; 11:e044005. doi: 10.1136/bmjopen-2020-044005
