# Supplementary material for: Taking charge of eczema self-management: a qualitative interview study with young people with eczema
Source: BMJ Open. 2021 Jan 6;11(1):e044005. doi: 10.1136/bmjopen-2020-044005 (PMC7789449; doi:10.1136/bmjopen-2020-044005)
Supplement: Supplementary data [file bmjopen-2020-044005supp002.pdf]

Supplementary File 2 - Interview Schedule for Eczema Care Online Project

Introduction

We are trying to find out as much as possible about your experiences of eczema, how you look after your eczema and how you learnt about this, or anything else you wish you knew. There are **no right or wrong answers** to any of these questions – it is just about trying to find out as much as possible in your own words. Any questions before we start?

Eczema views and experiences/perceived causes

- Could you tell me a bit about what its like to have eczema?
  - How does it affect you at the moment?
- What do you think causes eczema/flare ups?

Views and experiences of eczema treatments/self-care

- What kind of things do you do to help your eczema?
  - What treatments do you use for your eczema?
  - What do you think of your treatments?
- Can you tell me what you find easy/difficult about [insert specific]?
  - Could you talk me through maybe a daily or weekly routine of when you apply/use [insert treatment]?
- Could you tell me more about how you use [insert treatment they use]?
  - At home, at school?
  - What is easy/difficult about this?

  - If self-managing, how are you finding looking after your eczema? Or how would you feel about looking after your eczema?

- Do you do anything else to help make your eczema better?
  - Can you tell me what you find easy/difficult about that?
  - Are there also things that you've bought in shops or over the counter treatments that you've tried?
  - How do you try to reduce scratching?
  - How do you try to manage triggers/irritants?
  - Have you ever had anything like bath oils or soap substitutes?
  - Have you ever used relaxation techniques?

Experiences of eczema information provision

- What kinds of information have you had on eczema? From where?
- What's it like talking to health professionals (i.e. GPs, nurses, pharmacists)

Views about Quality of Life

- How does eczema make you feel?
  - Do you have strategies? – tell me more.
- Does eczema have much of an impact at all on what you wear or fabrics that you might choose?
- What about other things that you might use on your body (for example deodorant or perfume)?

Views about websites/web-based interventions

- How would you feel about using a website for information and support on eczema?

- What sort of things would you like to see on the website?
- What do you think would be helpful or would have been helpful to you in the past?
- We may include videos about eczema. What do you think would be good/not good about videos on eczema?

That is all really useful, thank you. Is there anything that we haven't talked about that you would like to add?

Greenwell K, *et al. BMJ Open* 2021; 11:e044005. doi: 10.1136/bmjopen-2020-044005
