# Supplementary material for: Taking charge of eczema self-management: a qualitative interview study with young people with eczema
Source: BMJ Open. 2021 Jan 6;11(1):e044005. doi: 10.1136/bmjopen-2020-044005 (PMC7789449; doi:10.1136/bmjopen-2020-044005)
Supplement: Author's manuscript [file bmjopen-2020-044005.draft_revisions.pdf]

BMJ Open is committed to open peer review. As part of this commitment we make the peer review history of every article we publish publicly available.

When an article is published we post the peer reviewers' comments and the authors' responses online. We also post the versions of the paper that were used during peer review. These are the versions that the peer review comments apply to.

The versions of the paper that follow are the versions that were submitted during the peer review process. They are not the versions of record or the final published versions. They should not be cited or distributed as the published version of this manuscript.

BMJ Open is an open access journal and the full, final, typeset and author-corrected version of record of the manuscript is available on our site with no access controls, subscription charges or pay-per-view fees (http://bmjopen.bmj.com).

If you have any questions on BMJ Open's open peer review process please email info.bmjopen@bmj.com

BMJ Open

Taking charge of eczema self-management: a qualitative interview study with young people with eczema

Journal:	*BMJ Open*
Manuscript ID	bmjopen-2020-044005
Article Type:	Original research
Date Submitted by the Author:	19-Aug-2020

Complete List of Authors:	Greenwell, Kate; University of Southampton School of Psychology,<br>Ghio, Daniela ; University of Southampton Faculty of Medicine, Primary Care, Population Science and Medical Education,<br>Muller, Ingrid; University of Southampton Faculty of Medicine, Primary Care, Population Science and Medical Education,<br>Roberts, Amanda; University of Nottingham, Centre of Evidence-Based Dermatology<br>McNiven, Abigail ; University of Oxford, Nuffield Department of Primary Care Health Sciences<br>Lawton, Sandra; Rotherham NHS Foundation Trust, Dermatology Department<br>Santer, Miriam; University of Southampton Faculty of Medicine, Primary Care, Population Science and Medical Education

Keywords:	Eczema < DERMATOLOGY, PRIMARY CARE, QUALITATIVE RESEARCH

SCHOLARONE™
Manuscripts

I, the Submitting Author has the right to grant and does grant on behalf of all authors of the Work (as defined in the below author licence), an exclusive licence and/or a non-exclusive licence for contributions from authors who are: i) UK Crown employees; ii) where BMJ has agreed a CC-BY licence shall apply, and/or iii) in accordance with the terms applicable for US Federal Government officers or employees acting as part of their official duties; on a worldwide, perpetual, irrevocable, royalty-free basis to BMJ Publishing Group Ltd ("BMJ") its licensees and where the relevant Journal is co-owned by BMJ to the co-owners of the Journal, to publish the Work in this journal and any other BMJ products and to exploit all rights, as set out in our licence.

The Submitting Author accepts and understands that any supply made under these terms is made by BMJ to the Submitting Author unless you are acting as an employee on behalf of your employer or a postgraduate student of an affiliated institution which is paying any applicable article publishing charge ("APC") for Open Access articles. Where the Submitting Author wishes to make the Work available on an Open Access basis (and intends to pay the relevant APC), the terms of reuse of such Open Access shall be governed by a Creative Commons licence – details of these licences and which Creative Commons licence will apply to this Work are set out in our licence referred to above.

Other than as permitted in any relevant BMJ Author's Self Archiving Policies, I confirm this Work has not been accepted for publication elsewhere, is not being considered for publication elsewhere and does not duplicate material already published. I confirm all authors consent to publication of this Work and authorise the granting of this licence.

For peer review only

TITLE: Taking charge of eczema self-management: a qualitative interview study with young people with eczema

Kate Greenwell[1*], Daniela Ghio[2], Ingrid Muller[2], Amanda Roberts[3], Abigail McNiven[4], Sandra Lawton[5]; Miriam Santer[2]

[1]Centre for Clinical and Community Applications of Health Psychology, Faculty of Environmental and Life Sciences, University of Southampton, Southampton, UK; [2]Primary Care, Population Science and Medical Education, Faculty of Medicine, University of Southampton, Southampton, UK; [3]Centre of Evidence Based Dermatology, School of Medicine, University of Nottingham, Nottingham, UK; [4]Nuffield Department of Primary Care Health Sciences, Radcliffe Observatory Quarter, Woodstock Road, Oxford, OX2 6GG, UK; [5] The Rotherham NHS Foundation Trust, Rotherham, UK.

***Corresponding Author:** Kate Greenwell, University of Southampton, Highfield Campus, Southampton, SO17 1BJ, UK; Email: K.Greenwell@soton.ac.uk

Word counts: 4000/4000 words

For peer review only

For peer review only - http://bmjopen.bmj.com/site/about/guidelines.xhtml

ABSTRACT

Objectives: To explore young people's experiences of eczema self-management and interacting with health professionals.

Design: Secondary qualitative data analysis of data sets from two semi-structured interview studies. Data was analysed using inductive thematic analysis.

Setting: Participants were recruited from UK primary care, dermatology departments, and a community-based sample (e.g. patient representative groups, social media).

Participants: Data included 28 interviews with young people with eczema aged 13-25 years (Mean age = 19.5 years; 20 female).

Results: Although topical treatments were generally perceived as effective, young people expressed doubts about their long-term effectiveness and concerns around the safety and an overreliance on topical corticosteroids. Participants welcomed the opportunity to take an active role in their eczema management, but new roles and responsibilities also came with initial apprehension and challenges, including communicating their treatment concerns and preferences with health professionals, feeling unprepared for transition to an adult clinic, and obtaining treatments. Decisions regarding whether to engage in behaviours that would exacerbate their eczema (e.g. irritants/triggers, scratching) were influenced by young people's beliefs regarding negative consequences of these behaviours, and perceived control over the behaviour and its negative consequences.

Conclusions: Behaviour change interventions must address the treatment concerns of young people and equip them with the knowledge, skills and confidence to take an active role in their own eczema management.

For peer review only - http://bmjopen.bmj.com/site/about/guidelines.xhtml

For peer review only

ARTICLE SUMMARY

Strengths and limitations of this study

- Our findings provided a more in-depth understanding of young people's unique experiences of eczema self-management and interactions with health professionals, which are largely not understood.
- Our in-depth dataset included diverse views from young people across a wide age range and from different ethnic groups.
- Although researchers from the primary research were involved in the data analysis, secondary analysis can overlook the socio-cultural-political context under which the research was originally conducted.
- For one interview study, sections of the dataset were to be published online, which meant that some participants may have been particularly motivated and may give socially desirable answers.

For peer review only - http://bmjopen.bmj.com/site/about/guidelines.xhtml

INTRODUCTION

Eczema is a common skin condition that can have a significant effect on an individuals' quality of life, mainly due to persistent itching and pain, sleep disturbance, and emotional distress.[1,2] Although eczema typically starts in infancy and is expected to resolve by late childhood, for many, symptoms can persist into adolescence and adulthood.[3] Eczema management focuses on the regular use of emollients to retain the skin's barrier function; treating flare-ups using topical corticosteroids (TSC); identification and avoidance of irritants/triggers; and avoiding scratching, which can further exacerbate eczema symptoms.[4]

Self-management can be particularly challenging during adolescence and early adulthood. Young people must take on a more active role in their eczema management, a role that was previously the primary responsibility of their families. They experience new demands, including understanding and adhering to complex treatment regimens; financing, acquiring, and testing effective medical treatments; interacting with health professionals; and negotiating healthcare systems. Such demands can serve to disrupt their health and well-being, and may lead to non-adherence and ineffective resource use.[5–7]

The unique self-management challenges and preferences of this group are largely not understood.[8] A recent systematic review found that most qualitative studies sampled young people with eczema alongside adults, children, or people with other skin conditions, making it difficult to tease out the unique experiences of this group.[8] The current study is the third from our research team to explore young people's experiences of eczema and its management. The previous two studies focused on young people's adaptation to eczema and its psychosocial impact.[3,9] This study aims to explore young people's experiences of eczema self-management and interacting with health professionals. This study will go beyond previous qualitative studies with young people[7] by exploring perceived challenges around

For peer review only - http://bmjopen.bmj.com/site/about/guidelines.xhtml

other self-management behaviours (i.e. avoiding irritants and triggers, reducing scratching), as well as topical treatments and, crucially, their experiences of taking an active role in eczema management.

METHODS

Design and data collection

The study design was a secondary analysis of qualitative interview data, drawing upon two data sources.

SKINS project: This project aimed to explore young people's experiences of living with common skin conditions (eczema, acne, psoriasis, alopecia) and share these on healthtalk.org. Participants were recruited from health settings, patient representative groups, educational institutions, and social media. Maximum variation sampling was used to recruit a range of demographics.[10] Twenty-four of the 97 interviews were with young people (aged 17-25 years) with eczema and all but one consented for their data to be used for this secondary analysis. Semi-structured interviews were carried out by AMcN (qualitative health researcher) between October 2014 and December 2015. They took place at the participants' homes or alternative meeting places, and lasted up to two hours. Interview topics included views about eczema treatment and management; experiences of healthcare; and advice for others with eczema and health professionals (Supplementary File 1). Interviews were either audio or video recorded and transcribed verbatim.

Eczema Care Online (ECO) project: As part of a wider project to develop an online intervention for young people with eczema, a survey and semi-structured interviews were carried out with this group. Interviews recruited younger adolescents (13-16 years) who were not captured in the SKINS sample. Sixteen GP practices and three hospitals across Wessex provided an invitation letter, participant information sheet, and survey to 1123 young people

For peer review only - http://bmjopen.bmj.com/site/about/guidelines.xhtml

with eczema (aged 13-25). Fifty-five young people agreed to take part in the interviews, 12 were 13-16 years old. Participants were purposively sampled to ensure a range of ages and a balance of genders. Five young people took part in the interviews. Those who did not take part in the interview either could not be contacted by telephone or were not selected for purposive sampling.

DG (expertise in paediatric psychology and qualitative research) interviewed participants at their homes during March-May 2018 lasting 22-50 minutes. Participants' parents were present for three of the interviews. Interview topics included views and experiences of eczema, treatments, self-management, and information and support for eczema (Supplementary File 2). Interviews were audio-recorded and transcribed verbatim.

Of the 28 participants overall, 20 were female and eight male. Participants had a mean age of 19.5 years and had eczema from 8 months to all their life (Table 1). The SKINS project was approved by Berkshire NRES Committee South Central, and the ECO data collection and both analyses were approved by Wales REC 7 Ethics Committee (REC 17/WA/0329).

Table 1 Demographics of participants from the two data sources

	Frequency	*n* (%)
**SKINS Project**		
Gender		
Male	6	(26)
Female	17	(74)
Age (years)		
16 – 18	4	(17)
19 – 21	10	(43)
22 – 24	9	(39)
Ethnicity (self-identified)		
White British	13	(57)

For peer review only - http://bmjopen.bmj.com/site/about/guidelines.xhtml

British Indian	3	(13)
Bangladeshi	2	(9)
Pakistani	2	(9)
Indian	1	(4)
Mixed - Black Caribbean and White	1	(4)
Mixed - White and Algerian	1	(4)
White Hungarian	1	(4)
Duration of condition (years)		
All life (diagnosis before first birthday)	16	(69)
11 to 21 years (diagnosis in toddler years)	4	(17)
5 to 7 years (diagnosis in adolescence years)	2	(8)
Up to 1 year (diagnosis in young adult years)	1	(4)
**Eczema Care Online Project**		
Gender		
Male	2	(40)
Female	3	(60)
Age (years)		
13 – 14	2	(40)
15 – 16	3	(60)
Duration of condition (years)		
All life (diagnosis before first birthday)	4	(80)
3 years (diagnosis in adolescence years)	1	(10)
Eczema Severity (self-identified)		
Moderate	3	(60)
Moderate/severe	1	(20)
Mild	1	(20)

Patient and public involvement

Our patient collaborator (AR) has eczema and children with eczema, and helps run a support group for carers of children with eczema. She was a co-applicant on the research grant

For peer review only - http://bmjopen.bmj.com/site/about/guidelines.xhtml

application, helping to identify the research topic and develop research questions. She attended regular project meetings, discussed and provided feedback on our interpretations of the findings, and provided feedback on the ECO study materials and this manuscript. She continues to help us to disseminate our research findings among her wide-reaching patient networks and via social media. Two young people reviewed the ECO participant information sheet to check comprehension. For the SKINS project, 8 PPI representatives (including young people with eczema, parents, and skin charity members), attended advisory group meetings, and reviewed the interview schedule and summaries of the findings.

Data analysis

Both data sets were analysed together using inductive thematic analysis[11] and data handling facilitated using NVivo 12 Pro. DG carried out initial coding on the entire SKINS dataset, categorising the data into various topics (e.g. barriers to emollients, experiences with health professionals). A coding manual was created and discussed with KG and IM (both health psychologists experienced in qualitative research) and MS (GP experienced in qualitative research). KG re-analysed all codes relevant to this study aim, coded the ECO data, and updated the coding manual accordingly. Disconfirming cases were identified and final themes were reviewed and agreed by all co-authors. Pseudonyms were given to all participants.

RESULTS

The five themes and the key findings under each theme are summarised in Figure 1.

Beliefs about the effectiveness of topical treatments

Generally, young people believed that their topical treatments (emollients and topical corticosteroids [TCS]) were effective. However, many described experiences whereby certain treatments had stopped working with time or caused new side effects (e.g. burning). This led

For peer review only - http://bmjopen.bmj.com/site/about/guidelines.xhtml

them to change emollient or TCS, apply increasingly more treatment to get the same effect, try a stronger TCS, or try a different type of treatment (e.g. phototherapy, immunosuppressants). Some participants attributed the reduced effectiveness of the treatments over time to their skin adapting to treatments (mainly emollients).

> *Generally it's [a new emollient] quite effective for a number of months and then that kind of tails off as either your body gets used to having that specific moisturiser and kind of taking it in [to the skin] too quickly and then it ends up getting dry again […] But switching around [emollients], generally, does make a difference.* (Mali, male, 23 years old, eczema for 20 years)

A minority reported how they had tried certain topical treatments (mainly TCS) that made their eczema worse, and a few did not find topical treatments made a difference to their eczema. A few participants stopped using emollients when their eczema was better or used them only when their eczema was problematic: *"I'm sort of taking the 'if it isn't broke don't fix it' route with my skin."* (Eevi, female, 22 years old, eczema since birth).

Concerns about topical treatments

Many young people expressed a general caution or hesitancy around using TCSs, listing side-effects (e.g. burning) or long-term effects they had experienced or had heard about (e.g. skin thinning, skin aging, impairing your immune system). For some, this cautiousness was about acknowledging the importance of using TCSs correctly and avoiding excessive and unnecessary use, whereas others actively avoided using TCSs or only used them when their eczema was very bad. Two participants expressed concerns that TCSs might cause eczema to *"spread"* or move around the body:

> *I feel that steroids sort of suppress it [eczema] and it crops up somewhere else…I want to get rid of the whole thing, not just have this internal thing inside me that will*

For peer review only - http://bmjopen.bmj.com/site/about/guidelines.xhtml

just crop up when and where because I'm not using something to suppress it. (Iris, female, 20 years old, eczema for 16 years)

Some participants were worried about becoming *"reliant"* on TCSs, having to use more, or increasingly stronger, TCSs or having to use them indefinitely. Many of their TCS concerns had originated from stories about potential side effects they had read or heard from others.

[I] met a girl who said that they [TCS] had the capacity to change your sexuality…so since then I've been really nervous [to use them]. (Hannah, female, 20 years old, eczema for 16 years)

[I] read about a lady...she used steroids for such a long time that it's definitely sort of had an adverse sort of reaction to her body...her hair's thinned and her…nails are brittle. (Dua, female, 21 years old, eczema for 20-21 years)

Such concerns about side-effects led some participants to switch to using more *"natural"* products to help eczema, sometimes against or at least without consulting medical advice. Natural (or homemade) products were viewed as *"healthy"*, described as containing no or fewer chemicals, and less likely to cause side-effects.

I wanted to stay away from that [potent TCS] because…I like naturally occurring products…my dermatologist…wasn't too happy for me to go down the route without steroids I think because…it does have a really high success rate. I won't deny that. (Iris, female, 20 years old, eczema for 16 years)

Compared to TCS, young people expressed fewer concerns regarding the safety of emollients. A few participants explained how emollients can *"block"* their skin and one expressed concerns about the flammability of emollients containing paraffin.

For peer review only - http://bmjopen.bmj.com/site/about/guidelines.xhtml

Participants found that topical treatments, specifically emollients that are applied regularly in large amounts, can be unpleasant in texture and/or smell, can make it difficult to use certain object (such as a pen), can rub off onto other items, such as your clothes or bedding, can make you feel hot, sweaty or *"dirty"*, and can sting when applied. Some avoided applying creams in public as they did not want others to *"find out"* they had eczema. They worried that others could smell their treatments, or that people would not want to touch them (e.g. partners holding their hand) if they had creams on. However, some explained how such worries reduced as they got older and started to care less about the others' opinions.

> *My colleagues often see me moisturising at my desk. They don't mention it [laughs] And if people think it's a bit weird, like I feel bad, but also like it's so uncomfortable when you can't moisturise.* (Willow, female, 23 years old, eczema since birth)

Burden of eczema self-management

Participants explained how burdensome eczema self-management could be. Applying topical treatments, identifying and avoiding irritants/triggers (especially as there are potentially so many), resisting the urge to scratch, and the 'trial and error' process of finding effective and acceptable (e.g. does not sting) treatments were described to be time-consuming, effortful, frustrating, and sometimes expensive.

> *It was quite a repetitive experience of just back and forth and back and forth. You'd explain [to the doctor], "oh okay well it's not working", like "okay let's, let's higher the dosage. Or let's try and find some steroids in", it just would not work.* (Raashid, male, 24 years, eczema since birth)

> *Some days you just can't be bothered. If you're out with friends, you know it's time to put on your cream; you would ignore it and during that night, I'd sometimes suffer the*

For peer review only - http://bmjopen.bmj.com/site/about/guidelines.xhtml

consequences…and I was scratching and itching all the time. (Qamar, male, 18 years old, eczema for 11 years)

As can be seen in the above quote, treatment burdens led some young people to skip treatments, especially when they had little time or energy.

Overcoming barriers to eczema self-management

Young people listed several practical barriers to eczema self-management and shared strategies they found helpful for overcoming these. Participants found it challenging to use their topical treatments (mainly emollients) when outside of the home. Specifically, it was difficult to use creams during class or take them when travelling abroad on a plane (due to luggage restrictions); or apply them if they worked in a public-facing job (e.g. retail or waiter). Other barriers to accessing treatments included running out of treatments, being unable to get a doctor's appointment to request more treatment (especially if they are living away from home and need to register with a GP), or treatment brands becoming discontinued. Participants found it helpful to keep sample-sized emollients or emollients decanted into smaller containers with them (e.g. in their bag) or around the house, to apply emollient before and after they leave the house, to stock up on treatments to avoid running out, and to be able to buy treatments without a prescription.

Young people explained how it could be difficult to remember to apply topical treatments and emphasised the importance of having a routine for applying treatments (especially their emollients), applying their creams at the same time each day, or making time to apply the creams: *"The biggest thing for fighting eczema is routine…do something that you think is working and do it regularly…don't be disorganised with it."* (Zaahira, female, 21 years old, eczema since birth).

For peer review only - http://bmjopen.bmj.com/site/about/guidelines.xhtml

Although young people acknowledged the importance of avoiding irritants and triggers, they explained that it could be difficult to control your environment, for example, staying at a friend's house, or in shared accommodation or an office environment. There were also external factors that they perceived to be unavoidable or outside of their control, such as the weather or stress.

Many participants commented on the cost of topical treatments, and cosmetics or washing products (e.g. shower gels, laundry detergents) that do not cause flare-ups. When they were younger, participants' treatments were free on the NHS, but many had to pay for their treatments from the age of 18. This cost prevented some from getting more treatment or led them to buy non-prescription treatments instead (e.g. over the counter medications). Participants particularly resented the cost when buying treatments or products that were ineffective or made their eczema worse.

> *I don't want to go to the doctors and I don't want to get more prescriptions because it just costs so much…once I had to fork out sort of about £36 worth of medications just for one particularly bad flare-up…and then if it doesn't work you're like, 'What, I spent £40 on nothing'.* (Dua, female, 21 years old, eczema for 20-21 years)

Some participants explained how they were still eligible for free prescriptions or obtained prescription prepayment certificates, which helped with their prescription costs.

Taking control of your eczema self-management

Young people shared their experiences of taking control of their own eczema management. They explained how, when they were a child, their parents did the majority of their eczema management. As they became a teenager, they started taking greater responsibility for, and playing a more active role in, their eczema care. Generally, this transition was welcomed by

For peer review only - http://bmjopen.bmj.com/site/about/guidelines.xhtml

participants and seen as a positive step towards becoming independent, a goal that was important to them.

> *As a child, I wasn't managing [eczema] at all, I'd let my parents do it [applying cream] whenever they felt like it needed being doing. But I wouldn't be applying my cream…And as teen, I realised that it was my problem and that I should be doing it and obviously as a teen you want your independence. You don't want your parents like stripping you off and covering you in bandages and cream.* (Jamila, female, 20 years old, eczema since birth)

> *My mum has always wanted to come with me to my appointments when I was older. But I've always said, "I want to do it on my own because I want to speak to my doctor." Cos [laughs] she had a habit when I was younger of coming with me to the doctor and…deciding what was being done for me.* (Iris, female, 20 years old, eczema for 16 years)

Like Jamila in the quote above, young people's drive for independence was prompted by a realisation that eczema management was their *"problem"* (rather than their parents' or health professionals') and they would have to live with the consequences of poorly managed eczema. For some, independence was important for maintaining privacy and avoiding their parents seeing them as vulnerable or distressed.

In contrast, some young people highlighted initial apprehension or challenges with these new roles and responsibilities, including moving to an adult clinic, having to do more things for yourself, and taking an active role in their medical consultations.

> *I'm not really looking forward to that because...they don't tell you what to do apparently in the adult clinic...you have to be by yourself in the adult clinic so you*

For peer review only - http://bmjopen.bmj.com/site/about/guidelines.xhtml

have to…pay more attention and they're not going to help me out as much as the children's nurse would. (Adi, male, 16 years old, eczema for 6.5-7 years)

Although young people were keen to take an active role, some found it intimidating to talk to health professionals or question their suggested treatments.

When I go to the doctors I feel like they're the person in charge so I can't really sort of be like, you know, "I know that this suits me best"…I think you do get intimidated…they sort of just give you like, you know that doctor tone, they're just a little bit better than you…and you're like, 'OK I guess you guys are right but [pause]'. (Dua, female, 21 years old, eczema for 20-21 years)

Many participants experienced times when their doctors or pharmacists were reluctant to prescribe their preferred treatments or refer them to a dermatologist. Conversely, some participants shared positive experiences of obtaining their preferred treatments from their health professionals and praised them for listening to them and their willingness to try different treatments. Young people stressed the importance of doing your own research around treatments (e.g. looking online), being *"confident"* or *"insistent"* when communicating your needs and preferences to health professionals, and writing a list of things to discuss beforehand.

Despite their changing role, the young people's families were still an important source of support for them: driving them to appointments, providing advice on eczema management, helping them apply topical treatments in difficult to reach areas, helping them understand what the doctor said, and speaking on their behalf at medical appointments when they felt unable to.

When deciding whether to engage in specific self-management behaviours, participants considered whether doing so was worth the potential impact it would inevitably have on their

For peer review only - http://bmjopen.bmj.com/site/about/guidelines.xhtml

eczema. For example, decisions around avoiding irritants/triggers were influenced by how difficult it was to avoid (e.g. if it was their favourite food, if everyone around them is drinking alcohol or eating a certain food), their belief about how much it would make their eczema worse, and whether any action could be taken to minimise these consequences (e.g. by applying emollients or taking antihistamines afterwards).

> *If I cut out lactose I don't think that it would make that much of a difference, so I might as well just eat the cheese and be happy.* (Eevi, female, 22 years old, eczema since birth)

> *I'd probably just carry on doing them [sports] and just sort of live with the consequences so, as long as you…go and have a shower afterwards and…cool down…if I'm having fun doing something then I'm not going to stop just cos of eczema.* (Lanie, female, 24 years old, eczema for 20+ years)

Participants were aware of the negative consequences of scratching, however, many found it difficult to avoid scratching completely. This was because a lot of their scratching was done sub-consciously, it was a habit, many of the self-management strategies (e.g. tapping) were ineffective, or the relief of scratching was rewarding: *"I'm terrible for scratching. I cannot stop myself. It's like an addiction cos it relieves such a horrible sensation all round my body."* (Iris, female, 20 years old, eczema for 16 years).

DISCUSSION

Our findings provided a more in-depth understanding of young people's unique experiences of eczema self-management. Specifically, the challenges they face when learning how to take an active role in their self-management: an issue that has not been explored in previous research. Although the new roles and responsibilities around eczema management were

For peer review only - http://bmjopen.bmj.com/site/about/guidelines.xhtml

generally welcomed by young people, they also brought initial apprehension and challenges. These findings mirrored previous research highlighting that young people often feel unprepared when transitioning from paediatric to adult care.[12,13]

Participants in this study found it hard to communicate their needs and treatment concerns to health professionals, which meant that, at times, they left consultations feeling like they were not listened to or were dissatisfied with the recommended course of action. Our previous qualitative study with this group found that young people with eczema have a strong need to feel understood and 'taken seriously' by their health professionals.[9] The more confident and competent young people feel in communicating their needs to health professionals, the more understood and validated they are likely to feel. There is a need for health professionals to acknowledge and encourage young people's desire to become active contributors to their own health care, treating them as an equal partner in their care, while recognising that this new role may be initially daunting for some.[14]

Our study explored how young people decide whether to engage in behaviours that would exacerbate their eczema (e.g. irritants/triggers, scratching). Previous research has focussed predominantly on the perceived benefits and costs of topical treatments.[8] Young people's decisions were influenced by their beliefs regarding the negative consequences of the behaviours, and their perceived control over the behaviour and its negative consequences. People with eczema have a desire to live a 'normal' life and not allow their eczema management impact on their everyday lives.[8,9] Behaviour change interventions can help promote self-management and, as argued by the self-determination theory, such interventions are more effective if they enhance an individual's need for autonomy and minimise conscious effort and lifestyle disruption.[15–17] It is important to support young people to decide which of these behaviours can be avoided and how to minimise the consequences of unavoidable irritants/triggers, focusing on what is positive and possible.[14]

For peer review only - http://bmjopen.bmj.com/site/about/guidelines.xhtml

The Burden of Treatment Theory[18] provides a useful framework for interpreting our study findings and making implications for practice. It argues that health interventions can minimise treatment burdens by building an individual's cognitive and material capacity to perform self-management tasks. In the context of our findings, this may involve building a young person's knowledge and skills required for making informed treatment decisions and weighing up the benefits and costs of self-management behaviours. For example, the financial cost of treatments is a common barrier amongst young people.[8] To build their material capacity, health professionals should acknowledge young people's concerns around the costs and educate them regarding any financial support that is available to them. The Burden of Treatment Theory argues that interventions should build and strengthen 'relational networks' (i.e. social networks, health professionals) around patients, and equip them to effectively navigate healthcare systems. Eczema interventions should build young people's confidence and communication skills and support them to make decisions, so they can direct their own care and feel prepared when transitioning to adult clinics.[14]

There are several limitations to this study. Secondary qualitative analysis has been criticised for not considering the socio-cultural-political context under which the primary research was conducted.[19] However, to minimise such decontextualisation and avoid any misinterpreted data, the interviewers of both data sets were co-authors (DG, AMcN). As sections of the transcriptions and, with participant consent, audio and video clips from the SKINS interviews were to be published online, participants from this dataset may have been particularly motivated and likely to give socially desirable answers, yet findings are congruent with other dataset.[7,8,12,13]

In conclusion, our findings highlight key self-management challenges faced by young people with eczema. Behaviour change interventions must address the treatment concerns of this

For peer review only - http://bmjopen.bmj.com/site/about/guidelines.xhtml

group and equip them with the knowledge, skills and confidence to take an active role in their eczema management.

ACKNOWLEDGEMENTS

We are grateful to the Health Experiences Research Group (University of Oxford) and Healthtalk.org for data sharing, and the young people who took part in this research. This manuscript presents independent research funded by the National Institute for Health Research (NIHR). The views expressed are those of the authors and not necessarily those of the NHS, the NIHR or the Department of Health and Social Care.

COMPETING INTERESTS

The authors have no competing interests to declare.

FUNDING

This study was funded by the National Institute for Health Research (NIHR) Programme Grants for Applied Research (RP-PG-0216-20007). Data collection for the SKINS project was funded by NIHR under its Research for Patient Benefit scheme (PB-PG-0213-30006).

CONTRIBUTOR STATEMENT

All authors designed the study. DG and AMcN was responsible for recruitment and data collection. KG led on the data analysis and drafted the manuscript, with support from DG, MS, IM. All authors critically reviewed the manuscript, contributing important intellectual content and approved the final manuscript.

DATA SHARING STATEMENT

The data that support the findings of this study are available from the corresponding author upon reasonable request.

For peer review only - http://bmjopen.bmj.com/site/about/guidelines.xhtml

REFERENCES

1 Ring J, Zink A, Arents BWM, *et al.* Atopic eczema: Burden of disease and individual suffering - results from a large EU study in adults. *J Eur Acad Dermatology Venereol* 2019;33:1331–40. doi:10.1111/jdv.15634

2 Maksimović N, Janković S, Marinković J, *et al.* Health-related quality of life in patients with atopic dermatitis. *J Dermatol* 2012;39:42–7. doi:10.1111/j.1346-8138.2011.01295.x

3 Ghio D, Muller I, Greenwell K, *et al.* 'It's like the bad guy in a movie who just doesn't die': A qualitative exploration of young people's adaptation to eczema and implications for self-care. *Br J Dermatol* 2020;182:112–8. doi:10.1111/bjd.18046

4 National Institute for Health and Care Excellence (NICE). Atopic eczema in under 12s: diagnosis and management (Clinical guideline 57). 2007. https://www.nice.org.uk/guidance/cg57 (accessed 17 Aug 2020).

5 Sav A, King MA, Whitty JA, *et al.* Burden of treatment for chronic illness: A concept analysis and review of the literature. *Heal Expect* 2015;18:312–24. doi:10.1111/hex.12046

6 Eton D, Ramalho de Oliveira D, Egginton J, *et al.* Building a measurement framework of burden of treatment in complex patients with chronic conditions: A qualitative study. *Patient Relat Outcome Meas* 2012;3:39–49. doi:10.2147/prom.s34681

7 Kosse RC, Bouvy ML, Daanen M, *et al.* Adolescents' perspectives on atopic dermatitis treatment-experiences, preferences, and beliefs. *JAMA Dermatology* 2018;154:824–7. doi:10.1001/jamadermatol.2018.1096

8 Teasdale E, Muller I, Sivyer K, *et al.* Views and experiences of managing eczema:

For peer review only - http://bmjopen.bmj.com/site/about/guidelines.xhtml

systematic review and thematic synthesis of qualitative studies. *Br J Dermatol* Published Online First: 12 June 2020. doi:10.1111/bjd.19299

9 Ghio D, Greenwell K, Muller I, *et al.* Psychosocial needs of adolescents and young adults with eczema: a secondary analysis of qualitative data to inform a behaviour change intervention. *Br J Health Psychol* in press.

10 McNiven A. 'Disease, illness, affliction? Don't know': Ambivalence and ambiguity in the narratives of young people about having acne. *Health (Irvine Calif)* 2019;23:273–88. doi:10.1177/1363459318762035

11 Braun V, Clarke V. Using thematic analysis in psychology. *Qual Res Psychol* 2006;3:77–101. doi:10.1191/1478088706qp063oa

12 Fegran L, Hall EOC, Uhrenfeldt L, *et al.* Adolescents' and young adults' transition experiences when transferring from paediatric to adult care: A qualitative metasynthesis. *Int J Nurs Stud* 2014;51:123–35. doi:10.1016/j.ijnurstu.2013.02.001

13 Betz CL, Lobo ML, Nehring WM, *et al.* Voices not heard: A systematic review of adolescents' and emerging adults' perspectives of health care transition. *Nurs Outlook* 2013;61:311–36. doi:10.1016/j.outlook.2013.01.008

14 National Institute for Health and Care Excellence (NICE). Transition from children's to adults' services for young people using health or social care services. 2016. https://www.nice.org.uk/guidance/ng43/resources/transition-from-childrens-to-adults-services-for-young-people-using-health-or-social-care-services-pdf-1837451149765 (accessed 17 Aug 2020).

15 Deci EL, Ryan RM. The 'what' and 'why' of goal pursuits: Human needs and the self-determination of behavior. *Psychol Inq* 2000;11:227–68.

For peer review only - http://bmjopen.bmj.com/site/about/guidelines.xhtml

doi:10.1207/S15327965PLI1104

16 Ryan RM, Deci EL. Self-determination theory and the facilitation of intrinsic motivation, social development, and well-being. *Am Psychol* 2000;55:68–78. doi:10.1037/0003-066X.55.1.68

17 Yardley L, Morrison L, Bradbury K, *et al.* The person-based approach to intervention development: Application to digital health-related behavior change interventions. *J Med Internet Res* 2015;17:e30. doi:10.2196/jmir.4055

18 May CR, Eton DT, Boehmer K, *et al.* Rethinking the patient: Using Burden of Treatment Theory to understand the changing dynamics of illness. *BMC Heal Serv Res Serv Res* 2014;14:281. doi:10.1186/1472-6963-14-281

19 Ruggiano N, Perry TE. Conducting secondary analysis of qualitative data: Should we, can we, and how? *Qual Soc Work* 2019;18:81–97. doi:10.1177/1473325017700701

FIGURE LEGENDS

Figure 1 Visual representation of themes and key findings

For peer review only

For peer review only - http://bmjopen.bmj.com/site/about/guidelines.xhtml

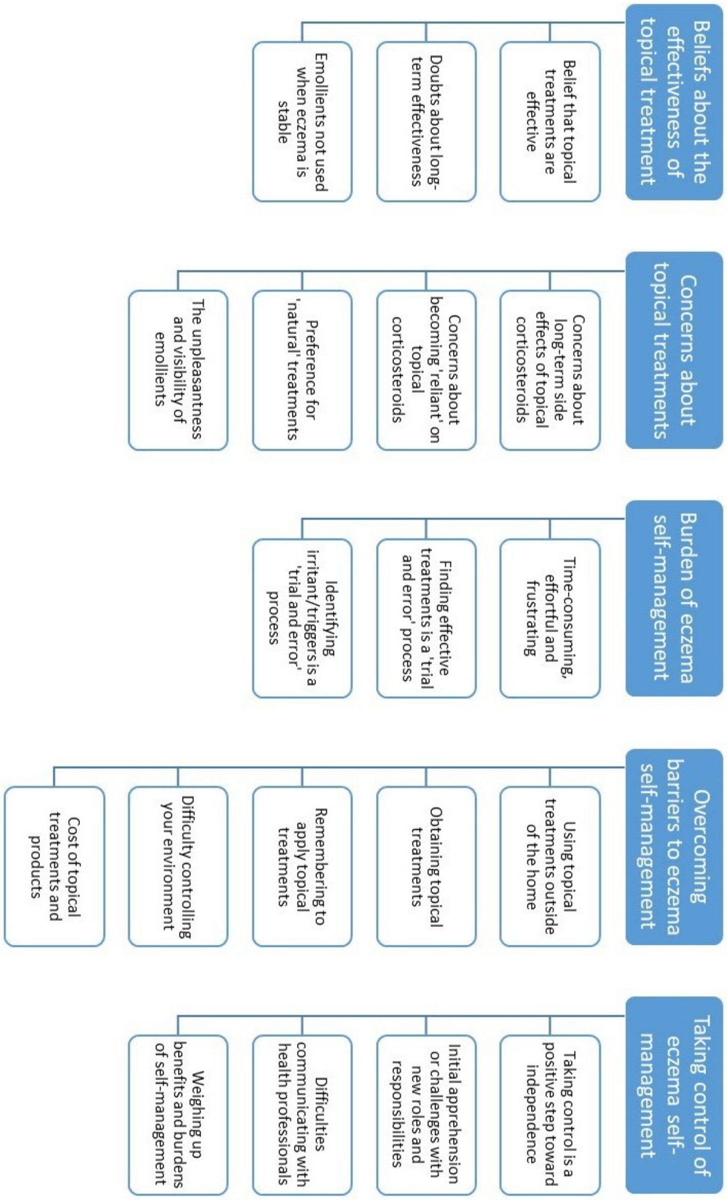

Visual representation of themes and key findings

Supplementary File 1 - Interview Schedule for SKINS Project

Part 1 of the interview: open ended/free-flow

- Could you tell me about your experiences of having [skin condition(s)]. You can say as a little or as much as you like about this.

Part 2 of the interview: follow-up and prompts

Realising that you had skin condition(s)

- What it was like when you first noticed that you might have a skin-related condition (or multiple skin-related conditions)?
- How long after you noticing/having symptoms did you talk to someone else about it?
- Have you been to see a doctor about your [skin condition(s)]? If so, at what stage did you first go?

Early knowledge/information about skin condition(s)

- What was it like when you were told you had [skin condition(s)]?
- Do you recall what, if anything, you knew about [skin condition(s)] before your diagnosis?
- After being diagnosed, did you look for other/more information elsewhere? If so, could you tell me about it?

Medical encounters/meetings

- Could I ask a bit more about the healthcare you've received for your [skin condition(s)]? So things like who do you see, where, how often?
- *If relevant:* What has it been like to move from a paediatric clinic to teenager and/or onto an adult clinic?

For peer review only - http://bmjopen.bmj.com/site/about/guidelines.xhtml

For peer review only

- Could you tell me about what happens when you go see [various health professionals]?

Treatments and managements of skin condition(s)

- Could you tell me about the treatments you have had in the past and any that you now do please?
- What were these treatments like? What did they involve?
- Did you have to make any decisions about these treatments? Like choosing between different options?
- Does anyone help you do these treatments? Or provide instructions about what you needed to do? Do they provide other kinds of practical help around your treatment?
- Are there some treatments or things to manage your [skin condition(s)] which are easier to do than others?
- Are there any which are particularly difficult or upsetting to do?
- Have there been any physical side effects to the treatments for your [skin condition(s)]?
- Have the treatments had any impact on the kinds of activities you can or would like to do?
- Have the treatments you've used had any other changes or prompted concerns for you?

Changes to the skin condition over time and in the anticipated future

- Could you run me through a typical day/week/month from the point of view of managing [skin condition(s)]?
- Have there been any changes over the time you've had [skin condition(s)] as to what the condition has been like (better, worse)? Either in relation to treatments or unexpectedly.

For peer review only

For peer review only - http://bmjopen.bmj.com/site/about/guidelines.xhtml

- Are there any more treatments planned or possible options for management in the future for you?

Everyday life as a young person with [skin condition(s)]

- Could you tell me what it is like being a young person with [skin condition(s)] (as opposed to a child or older adult with this)?
- *If relevant:* Do you think that [skin condition(s)] has an impact for you in terms of school/ college/university?
- *If relevant:* How about any impact in relation to employers (full-time, part-time)? This could be present, past and future/prospective employers.
- Are there any ways in which [skin condition(s)] plays a part in your friendships? This might include friendships at school/college/uni and/or at work but also beyond (including online)?
- Could I ask about any impacts of [skin condition(s)] on (potential and/or actual) romantic relationships?
- In your experience, does having [skin condition(s)] make a difference with regards to social situations or events you might attend? In a negative way or in a positive way?
- Are there any particular places you might go for which are affected in any way by [skin condition(s)]? For example, any places/spaces in particular that you would avoid? Or places where you feel more comfortable? Places you've gone to because of [skin condition(s)]?

Home life and [skin condition(s)]

- Are there any other aspects of your home life and lifestyle that you think having [skin condition(s)] affects? If so, how and why?
- What about any impact on your family [parents, siblings etc.] and/or those you live with [e.g. if in independent accommodation]?

For peer review only - http://bmjopen.bmj.com/site/about/guidelines.xhtml

'Body work' and body-image

- Does your [skin condition(s)] affect the things you can wear or otherwise put on your body/skin? For example cosmetics, make-up, clothes, piercings, tattoos, hair dying, depilation.

Subsequent information and support

- Could you tell me about any practical and/or emotional support you've found or received, either from individuals, organisations or websites?
- Are you aware of any support groups either for your condition or more generally for young people?

Sharing information/messages to others

- What would be your advice to other young people with [skin condition(s)]? For example, is there anything from your experience that you think they might be interested to know or might find helpful?
- Is there anything you wish you had known, or anything you still don't know and would like to, about [skin condition(s)]? Are there things you'd like to know about others experiences?
- Based on your experience of [skin condition(s)], is there anything you think doctors and nurses should know? What would be your message to them?

Closing

- Is there anything else about your experience of [skin condition(s)] that I haven't covered but that you think might be important? Or would you like to add to something you've said previously?

For peer review only - http://bmjopen.bmj.com/site/about/guidelines.xhtml

Supplementary File 2 - Interview Schedule for Eczema Care Online Project

Introduction

We are trying to find out as much as possible about your experiences of eczema, how you look after your eczema and how you learnt about this, or anything else you wish you knew. There are **no right or wrong answers** to any of these questions – it is just about trying to find out as much as possible in your own words. Any questions before we start?

Eczema views and experiences/perceived causes

- Could you tell me a bit about what its like to have eczema?
  - How does it affect you at the moment?
- What do you think causes eczema/flare ups?

Views and experiences of eczema treatments/self-care

- What kind of things do you do to help your eczema?
  - What treatments do you use for your eczema?
  - What do you think of your treatments?
- Can you tell me what you find easy/difficult about [insert specific]?
  - Could you talk me through maybe a daily or weekly routine of when you apply/use [insert treatment]?
- Could you tell me more about how you use [insert treatment they use]?
  - At home, at school?
  - What is easy/difficult about this?

For peer review only - http://bmjopen.bmj.com/site/about/guidelines.xhtml

- If self-managing, how are you finding looking after your eczema? Or how would you feel about looking after your eczema?

- Do you do anything else to help make your eczema better?
    - Can you tell me what you find easy/difficult about that?
    - Are there also things that you've bought in shops or over the counter treatments that you've tried?
    - How do you try to reduce scratching?
    - How do you try to manage triggers/irritants?
    - Have you ever had anything like bath oils or soap substitutes?
    - Have you ever used relaxation techniques?

Experiences of eczema information provision

- What kinds of information have you had on eczema? From where?
- What's it like talking to health professionals (i.e. GPs, nurses, pharmacists)

Views about Quality of Life

- How does eczema make you feel?
    - Do you have strategies? – tell me more.
- Does eczema have much of an impact at all on what you wear or fabrics that you might choose?
- What about other things that you might use on your body (for example deodorant or perfume)?

Views about websites/web-based interventions

- How would you feel about using a website for information and support on eczema?

For peer review only

For peer review only - http://bmjopen.bmj.com/site/about/guidelines.xhtml

- What sort of things would you like to see on the website?
- What do you think would be helpful or would have been helpful to you in the past?
- We may include videos about eczema. What do you think would be good/not good about videos on eczema?

That is all really useful, thank you. Is there anything that we haven't talked about that you would like to add?

For peer review only

For peer review only - http://bmjopen.bmj.com/site/about/guidelines.xhtml

Greenwell et al. Taking charge of eczema self-management: a qualitative interview study with young people with eczema

COREQ checklist

No	Item	Guide questions/description	Comments	Location in manuscript
**Domain 1: Research team and reflexivity**				
Personal Characteristics				
1.	Interviewer/facilitator	Which author/s conducted the interview or focus group?	AMcN, DG	Page 5&6
2.	Credentials	What were the researcher's credentials? *E.g. PhD, MD*	KG – PhD, CPsychol. AMcN – PhD DG – PhD, CPsychol	-
3.	Occupation	What was their occupation at the time of the study?	KG – Health Psychologist & Senior Research Fellow AMcN – Qualitative Health Researcher DG – Research Fellow and Charted Psychologist	-
4.	Gender	Was the researcher male or female?	All female	-
5.	Experience and training	What experience or training did the researcher have?	All experienced qualitative postdoctoral researchers.	Page 5,6-8
Relationship with participants				
6.	Relationship established	Was a relationship established prior to study commencement?	Most participants were not known to the interviewers prior to recruitment, but two participants in the SKINS project were.	-
7.	Participant knowledge of the interviewer	What did the participants know about the researcher? e.g. *personal goals, reasons for doing the research*	SKINS - Participants were told that sections of the transcriptions and, with participant consent, audio and video clips from the interviews were to be published online. ECO – Participants were told that the interviews were to help develop a self-management website for young people.	Page 5,18
8.	Interviewer characteristics	What characteristics were reported about the interviewer/facilitator? e.g. *Bias,*	Interviewers and data coders was carrying out the research to inform health interventions, which	-

For peer review only

Greenwell et al. Taking charge of eczema self-management: a qualitative interview study with young people with eczema

No	Item	Guide questions/description	Comments	Location in manuscript
		assumptions, reasons and interests in the research topic	could be a potential source of bias. One of the interviewees had visible hand eczema during the interviews, which may have influenced how participants share their eczema experiences.	
**Domain 2: study design**				
Theoretical framework				
9.	Methodological orientation and Theory	What methodological orientation was stated to underpin the study? *e.g. grounded theory, discourse analysis, ethnography, phenomenology, content analysis*	Thematic analysis.	Page 8
Participant selection				
10.	Sampling	How were participants selected? *e.g. purposive, convenience, consecutive, snowball*	Maximum variation sampling/purposive sampling.	Page 5&6
11.	Method of approach	How were participants approached? e.g. *face-to-face, telephone, mail, email*	Mail, face-to-face, telephone, email, research advertisements via social media, patient representative groups, and NHS sites (e.g. posters in waiting rooms, packs handed out by health professionals).	Page 5&6
12.	Sample size	How many participants were in the study?	28	Page 6
13.	Non-participation	How many people refused to participate or dropped out? Reasons?	ECO – Of the 1123 young people approached, 55 agreed to take part in the interviews, 12 were 13-16 years old. Seven of these did not take part in the interview either could not be contacted by telephone or were not selected for purposive sampling.	Page 5&6

For peer review only - http://bmjopen.bmj.com/site/about/guidelines.xhtml

Greenwell et al. Taking charge of eczema self-management: a qualitative interview study with young people with eczema

No	Item	Guide questions/description	Comments	Location in manuscript
			SKINS – One interview participant did not provide consent for their data to be used.	
Setting				
14.	Setting of data collection	Where was the data collected? e.g. *home, clinic, workplace*	At the participants' homes or alternative meeting places.	Page 5&6
15.	Presence of non-participants	Was anyone else present besides the participants and researchers?	Relatives were occasionally present during the interviews.	Page 5&6
16.	Description of sample	What are the important characteristics of the sample? *e.g. demographic data, date*	Demographic information can be found in Table 1. Interviews were carried out during October 2014 and December 2015, and March-May 2018.	Table 1; page 5 & 6
Data collection				
17.	Interview guide	Were questions, prompts, guides provided by the authors? Was it pilot tested?	The interview schedules can be found in Appendix 1 and 2. The SKINS interview schedule was reviewed by PPI representatives.	Appendix 1&2.
18.	Repeat interviews	Were repeat interviews carried out? If yes, how many?	Only single interviews were carried out.	-
19.	Audio/visual recording	Did the research use audio or visual recording to collect the data?	Audio or video (SKINS only) recording.	Page 5&6
20.	Field notes	Were field notes made during and/or after the interview or focus group?	Field notes were made after all interviews.	-
21.	Duration	What was the duration of the interviews or focus group?	SKINS interviews lasted up to 2 hours; ECO interviews between 22-50 minutes	Page 5&6
22.	Data saturation	Was data saturation discussed?	No	-
23.	Transcripts returned	Were transcripts returned to participants for comment and/or correction?	SKINS participants were sent a copy of the transcripts and they could remove sections and correct or add details if they wished.	-

For peer review only

Greenwell et al. Taking charge of eczema self-management: a qualitative interview study with young people with eczema

No	Item	Guide questions/description	Comments	Location in manuscript
**Domain 3: analysis and findings**				
Data analysis				
24.	Number of data coders	How many data coders coded the data?	Two (KG & DG)	Page 8
25.	Description of the coding tree	Did authors provide a description of the coding tree?	No	-
26.	Derivation of themes	Were themes identified in advance or derived from the data?	Derived from the data (inductive analysis).	Page 8
27.	Software	What software, if applicable, was used to manage the data?	QSR's NVivo 12 Pro was used.	Page 8
28.	Participant checking	Did participants provide feedback on the findings?	SKINS participants were sent a copy of the transcripts and they could remove sections and correct or add details if they wished. PPI provided feedback on summaries of the findings.	Page 7-8
Reporting				
29.	Participant checking	Were participant quotations presented to illustrate the themes / findings? Was each quotation identified? e.g. *participant number*	Participant quotations are presented and each quotation is identified by a pseudonym and their gender, age and eczema duration is noted.	Page 9-16
30.	Data and findings consistent	Was there consistency between the data presented and the findings?	Yes	Page 8-16
31.	Clarity of major themes	Were major themes clearly presented in the findings?	Yes	Page 8-16; Figure 1
32.	Clarity of minor themes	Is there a description of diverse cases or discussion of minor themes?	Diverse cases are discussed.	Page 8-16.

For peer review only

BMJ Open

Taking charge of eczema self-management: a qualitative interview study with young people with eczema

Journal:	*BMJ Open*
Manuscript ID	bmjopen-2020-044005.R1
Article Type:	Original research
Date Submitted by the Author:	01-Dec-2020
Complete List of Authors:	Greenwell, Kate; University of Southampton School of Psychology,<br>Ghio, Daniela ; University of Southampton Faculty of Medicine, Primary Care, Population Science and Medical Education,<br>Muller, Ingrid; University of Southampton Faculty of Medicine, Primary Care, Population Science and Medical Education,<br>Roberts, Amanda; University of Nottingham, Centre of Evidence-Based Dermatology<br>McNiven, Abigail ; University of Oxford, Nuffield Department of Primary Care Health Sciences<br>Lawton, Sandra; Rotherham NHS Foundation Trust, Dermatology Department<br>Santer, Miriam; University of Southampton Faculty of Medicine, Primary Care, Population Science and Medical Education
**Primary Subject Heading**:	Dermatology
Secondary Subject Heading:	Qualitative research
Keywords:	Eczema < DERMATOLOGY, PRIMARY CARE, QUALITATIVE RESEARCH

SCHOLARONE™
Manuscripts

I, the Submitting Author has the right to grant and does grant on behalf of all authors of the Work (as defined in the below author licence), an exclusive licence and/or a non-exclusive licence for contributions from authors who are: i) UK Crown employees; ii) where BMJ has agreed a CC-BY licence shall apply, and/or iii) in accordance with the terms applicable for US Federal Government officers or employees acting as part of their official duties; on a worldwide, perpetual, irrevocable, royalty-free basis to BMJ Publishing Group Ltd ("BMJ") its licensees and where the relevant Journal is co-owned by BMJ to the co-owners of the Journal, to publish the Work in this journal and any other BMJ products and to exploit all rights, as set out in our licence.

The Submitting Author accepts and understands that any supply made under these terms is made by BMJ to the Submitting Author unless you are acting as an employee on behalf of your employer or a postgraduate student of an affiliated institution which is paying any applicable article publishing charge ("APC") for Open Access articles. Where the Submitting Author wishes to make the Work available on an Open Access basis (and intends to pay the relevant APC), the terms of reuse of such Open Access shall be governed by a Creative Commons licence – details of these licences and which Creative Commons licence will apply to this Work are set out in our licence referred to above.

Other than as permitted in any relevant BMJ Author's Self Archiving Policies, I confirm this Work has not been accepted for publication elsewhere, is not being considered for publication elsewhere and does not duplicate material already published. I confirm all authors consent to publication of this Work and authorise the granting of this licence.

For peer review only

TITLE: Taking charge of eczema self-management: a qualitative interview study with young people with eczema

Kate Greenwell[1*], Daniela Ghio[2], Ingrid Muller[2], Amanda Roberts[3], Abigail McNiven[4], Sandra Lawton[5]; Miriam Santer[2]

[1]Centre for Clinical and Community Applications of Health Psychology, Faculty of Environmental and Life Sciences, University of Southampton, Southampton, UK; [2]Primary Care, Population Science and Medical Education, Faculty of Medicine, University of Southampton, Southampton, UK; [3]Centre of Evidence Based Dermatology, School of Medicine, University of Nottingham, Nottingham, UK; [4]Nuffield Department of Primary Care Health Sciences, University of Oxford, Radcliffe Observatory Quarter, Woodstock Road, Oxford, OX2 6GG, UK; [5] The Rotherham NHS Foundation Trust, Rotherham, UK.

***Corresponding Author:** Kate Greenwell, University of Southampton, Highfield Campus, Southampton, SO17 1BJ, UK; Email: K.Greenwell@soton.ac.uk

Word counts: 4982 words

For peer review only

ABSTRACT

Objectives: To explore young people's experiences of eczema self-management and interacting with health professionals.

Design: Secondary qualitative data analysis of data sets from two semi-structured interview studies. Data was analysed using inductive thematic analysis.

Setting: Participants were recruited from UK primary care, dermatology departments, and a community-based sample (e.g. patient representative groups, social media).

Participants: Data included 28 interviews with young people with eczema aged 13-25 years (Mean age = 19.5 years; 20 female).

Results: Although topical treatments were generally perceived as effective, young people expressed doubts about their long-term effectiveness and concerns around the safety and an overreliance on topical corticosteroids. Participants welcomed the opportunity to take an active role in their eczema management, but new roles and responsibilities also came with initial apprehension and challenges, including communicating their treatment concerns and preferences with health professionals, feeling unprepared for transition to an adult clinic, and obtaining treatments. Decisions regarding whether to engage in behaviours that would exacerbate their eczema (e.g. irritants/triggers, scratching) were influenced by young people's beliefs regarding negative consequences of these behaviours, and perceived control over the behaviour and its negative consequences.

Conclusions: Behaviour change interventions must address the treatment concerns of young people and equip them with the knowledge, skills and confidence to take an active role in their own eczema management.

For peer review only - http://bmjopen.bmj.com/site/about/guidelines.xhtml

ARTICLE SUMMARY

Strengths and limitations of this study

- Our findings provided a more in-depth understanding of young people's unique experiences of eczema self-management and interactions with health professionals, which are largely not understood.
- Our in-depth dataset included diverse views from young people across a wide age range and from different ethnic groups.
- Although researchers from the primary research were involved in the data analysis, secondary analysis can overlook the socio-cultural-political context under which the research was originally conducted.
- For one interview study, sections of the dataset were to be published online, which meant that some participants may have been particularly motivated and may give socially desirable answers.

For peer review only - http://bmjopen.bmj.com/site/about/guidelines.xhtml

INTRODUCTION

Eczema is a common skin condition that can have a significant effect on an individuals' quality of life, mainly due to persistent itching and pain, sleep disturbance, and emotional distress.[1,2] Although eczema typically starts in infancy and epidemiology studies show that it typically improves or resolves by late childhood,[3,4] for many, symptoms can persist into adolescence and adulthood.[5] For most, eczema management focuses on the regular use of emollients to retain the skin's barrier function; treating flare-ups using topical corticosteroids (TSC); identification and avoidance of irritants/triggers; and avoiding scratching, which can further exacerbate eczema symptoms.[6] For those with moderate and severe eczema, management may also involve use of topical calcineurin inhibitors, bandages or medicated dressings, systemic therapy (immunosuppressants), and phototherapy (light therapy).[6]

Self-management can be particularly challenging during adolescence and early adulthood. Young people must take on a more active role in their eczema management, a role that was previously the primary responsibility of their families. They experience new demands, including understanding and adhering to complex treatment regimens; financing, acquiring, and testing effective medical treatments; interacting with health professionals; and negotiating healthcare systems. Such demands can serve to disrupt their health and well-being, and may lead to non-adherence and ineffective resource use.[7–9]

The unique self-management challenges and preferences of this group are largely not understood.[10] A recent systematic review found that most qualitative studies sampled young people with eczema alongside adults, children, or people with other skin conditions, making it difficult to tease out the unique experiences of this group.[10] The current study is the third from our research team to explore young people's experiences of eczema and its management. Each study involved a secondary data analysis of a large qualitative data set derived from two primary studies exploring young people's experiences of eczema, but

For peer review only - http://bmjopen.bmj.com/site/about/guidelines.xhtml

focused on a different aspect of these accounts. The first study explored perceptions about the nature of eczema (e.g. as an episodic long-term condition) and how these perceptions related to their self-care and adaptation to eczema.[11] The second study explored young people's experiences of eczema-related symptoms (both visible and invisible to others) to determine their psychosocial needs.[12] The current study aims to explore young people's experiences of eczema self-management and interacting with health professionals. This study will go beyond previous qualitative studies with young people[9] by exploring perceived challenges around other self-management behaviours (i.e. avoiding irritants and triggers, reducing scratching), as well as topical treatments and, crucially, their experiences of taking an active role in eczema management.

METHODS

Design and data collection

The study design was a secondary analysis of qualitative interview data, drawing upon two data sources. Although both studies had different aims, both used interview questions relevant to this study aim, exploring experiences of eczema treatments and management, and interactions with health professionals.

SKINS project: This project aimed to explore young people's experiences of living with common skin conditions (eczema, acne, psoriasis, alopecia) and share these on healthtalk.org. Participants were recruited from health settings, patient representative groups, educational institutions, and social media. Maximum variation sampling was used to recruit a range of demographics.[13] Twenty-four of the 97 interviews were with young people (aged 17-25 years) with eczema and all but one consented for their data to be used for this secondary analysis. Semi-structured interviews were carried out by AMcN (qualitative health researcher) between October 2014 and December 2015. They took place at the participants' homes or alternative meeting places, and lasted up to two hours. Interview topics included

For peer review only - http://bmjopen.bmj.com/site/about/guidelines.xhtml

For peer review only

views about eczema treatment and management; experiences of healthcare; and advice for others with eczema and health professionals (Supplementary File 1). Interviews were either audio or video recorded and transcribed verbatim, and checked by participants for accuracy. All three of our secondary data analysis studies drew upon this data source.

Eczema Care Online (ECO) project: As part of a wider project to develop an online intervention for young people with eczema, a survey and semi-structured interviews were carried out with this group. Interviews recruited younger adolescents (13-16 years) who were not captured in the SKINS sample. Sixteen GP practices and three hospitals across Wessex provided an invitation letter, participant information sheet, and survey to 1123 young people with eczema (aged 13-25). Fifty-five young people agreed to take part in the interviews, 12 were 13-16 years old. Participants were purposively sampled to ensure a range of ages and a balance of genders. Five young people took part in the interviews. Those who did not take part in the interview either could not be contacted by telephone or were not selected for purposive sampling. This data source was also used in one of our other secondary data analysis studies.[12]

DG (expertise in paediatric psychology and qualitative research) interviewed participants at their homes during March-May 2018 lasting 22-50 minutes. Participants' parents were present for three of the interviews. Interview topics included views and experiences of eczema, treatments, self-management, and information and support for eczema (Supplementary File 2). Interviews were audio-recorded and transcribed verbatim.

Of the 28 participants overall, 20 were female and eight male. Participants had a mean age of 19.5 years and had eczema for a period of 8 months to all their life (Table 1). The SKINS project was approved by Berkshire NRES Committee South Central, and the ECO data

For peer review only - http://bmjopen.bmj.com/site/about/guidelines.xhtml

collection and both analyses were approved by Wales REC 7 Ethics Committee (REC 17/WA/0329).

Table 1 Demographics of participants from the two data sources

	Frequency	*n* (%)
**SKINS Project**		
Gender		
Male	6	(26)
Female	17	(74)
Age (years)		
16 – 18	4	(17)
19 – 21	10	(43)
22 – 24	9	(39)
Ethnicity (self-identified)		
White British	13	(57)
British Indian	3	(13)
Bangladeshi	2	(9)
Pakistani	2	(9)
Indian	1	(4)
Mixed - Black Caribbean and White	1	(4)
Mixed - White and Algerian	1	(4)
White Hungarian	1	(4)
Duration of condition (years)		
All life (diagnosis before first birthday)	16	(69)
11 to 21 years (diagnosis in toddler years)	4	(17)
5 to 7 years (diagnosis in adolescence years)	2	(8)
Up to 1 year (diagnosis in young adult years)	1	(4)
**Eczema Care Online Project**		
Gender		
Male	2	(40)

For peer review only - http://bmjopen.bmj.com/site/about/guidelines.xhtml

For peer review only

Female	3	(60)
Age (years)		
13 – 14	2	(40)
15 – 16	3	(60)
Duration of condition (years)		
All life (diagnosis before first birthday)	4	(80)
3 years (diagnosis in adolescence years)	1	(10)
Eczema Severity (self-identified)		
Moderate	3	(60)
Moderate/severe	1	(20)
Mild	1	(20)

Patient and public involvement

Our patient collaborator (AR) has eczema and children with eczema, and helps run a support group for carers of children with eczema. She was a co-applicant on the research grant application, helping to identify the research topic and develop research questions. She attended regular project meetings, discussed and provided feedback on our interpretations of the findings, and provided feedback on the ECO study materials and this manuscript. She continues to help us to disseminate our research findings among her wide-reaching patient networks and via social media. Two young people reviewed the ECO participant information sheet to check comprehension. For the SKINS project, 8 PPI representatives (including young people with eczema, parents, and skin charity members) attended advisory group meetings and reviewed the interview schedule and summaries of the findings.

Data analysis

Both data sets were analysed together by following the six stages of Braun and Clarke's inductive thematic analysis[14] and data handling was facilitated using NVivo 12 Pro. DG carried out initial coding on the entire SKINS dataset, familiarising herself with the data and

For peer review only

For peer review only - http://bmjopen.bmj.com/site/about/guidelines.xhtml

generating initial codes that represented the various topics present across the data (e.g. barriers to emollients, experiences with health professionals). A coding manual was created, including descriptions of the codes and example quotes, and discussed with KG and IM (both health psychologists experienced in qualitative research) and MS (GP experienced in qualitative research). These three researchers used the manual to code two transcripts and changes to the coding manual were made following discussions. The revised coding manual was discussed with a PPI representative (AR) and the interviewer for the SKINS project (AMcN). It was agreed that the findings would be reported across three separate studies (the other two are reported elsewhere[11,12]).

For this study, KG reviewed the initial codes deemed relevant to the study aim, redefined them in line with these aims, searched for, reviewed and defined themes, and created a coding manual for this study. KG then familiarised herself with the ECO data, and coded the data, identifying any data that deviated from the SKINS data (e.g. any new codes or experiences) in the coding manual. Disconfirming cases were identified throughout the entire dataset. Writing up the thematic analysis in this manuscript helped finalise the analysis, enabling the final themes to be reviewed and agreed by all co-authors. Pseudonyms were given to all participants.

An important quality criteria in qualitative research is the adequacy of the sample size. This can be assessed by consideration of a study's 'information power' [15] and whether data saturation was reached.[16] Drawing on the guidelines on information power in qualitative interview studies,[15] our sample size for the secondary data analysis (n=28) was deemed adequate given the specificity of the study aim (self-management) and target group (young people), the large amount of data (in particular from the SKINS interviews which lasted up to two hours), and the likely high quality of dialogue from using experienced qualitative postdoctoral researchers. It was also comparable to other qualitative interview studies

For peer review only - http://bmjopen.bmj.com/site/about/guidelines.xhtml

exploring experiences among people with eczema[10]. When analysing the SKINS data, we felt that data saturation was reached as the later interviews did not identify any additional codes or diversely different experiences or views.[16] Although the analysis of the ECO project data did not identify any additional codes (i.e. code saturation was reached [16]), the analysts felt that additional interviews with this age group was needed to be confident that the issues facing this group are fully understood (i.e. meaning saturation was not reached for this group[16]).

RESULTS

The five themes and the key findings under each theme are summarised in Figure 1.

Beliefs about the effectiveness of topical treatments

Generally, young people believed that their topical treatments (emollients and topical corticosteroids [TCS], topical calcineurin inhibitors) were effective. However, many described experiences whereby certain treatments had stopped working with time or caused new side effects (e.g. burning). This led them to change emollient or TCS, apply increasingly more treatment to get the same effect, or try a stronger TCS. Some participants attributed the reduced effectiveness of the treatments over time to their skin adapting to treatments (mainly emollients).

> *I can use one moisturiser for six months, a year, and then my skin will get too used to it and I'll have to keep putting more on. Then I'll end up having to switch to a different kind of moisturiser just so I can get it under control again.* (Cassie, female, 15 years old, eczema since birth, ECO project)

> *Switching around [emollients], generally, does make a difference.* (Mali, male, 23 years old, eczema for 20 years, SKINS project)

For peer review only

A minority reported how they had tried certain topical treatments (mainly TCS) that made their eczema worse, and a few did not find topical treatments made a difference to their eczema. A few participants stopped using emollients when their eczema was better or used them only when their eczema was problematic: *"I'm sort of taking the 'if it isn't broke don't fix it' route with my skin."* (Eevi, female, 22 years old, eczema since birth, SKINS project).

Concerns about topical treatments

Many young people expressed a general caution or hesitancy around using TCS, listing side-effects (e.g. burning) or long-term effects they had experienced or had heard about (e.g. skin thinning, skin aging, impairing your immune system). For some, this cautiousness was about acknowledging the importance of using TCS correctly and avoiding excessive and unnecessary use, whereas others actively avoided using TCS or only used them when their eczema was very bad. Two participants expressed concerns that TCS might cause eczema to *"spread"* or move around the body:

> *I feel that steroids sort of suppress it [eczema] and it crops up somewhere else…I want to get rid of the whole thing, not just have this internal thing inside me that will just crop up when and where because I'm not using something to suppress it.* (Iris, female, 20 years old, eczema for 16 years, SKINS project)

Some participants were worried about becoming *"reliant"* on TCS, having to use more, or increasingly stronger, TCS or having to use them indefinitely. Many of their TCS concerns had originated from stories about potential side effects they had read or heard from others.

> *[I] met a girl who said that they [TCS] had the capacity to change your sexuality…so since then I've been really nervous [to use them].* (Hannah, female, 20 years old, eczema for 16 years, SKINS project)

For peer review only - http://bmjopen.bmj.com/site/about/guidelines.xhtml

[I] read about a lady...she used steroids for such a long time that it's definitely sort of had an adverse sort of reaction to her body...her hair's thinned and her…nails are brittle. (Dua, female, 21 years old, eczema for 20-21 years, SKINS project)

Such concerns about side-effects led some participants to switch to using more *"natural"* products to help eczema, sometimes against or at least without consulting medical advice. Natural (or homemade) products were viewed as *"healthy"*, described as containing no or fewer chemicals, and less likely to cause side-effects.

I wanted to stay away from that [potent TCS] because…I like naturally occurring products…my dermatologist…wasn't too happy for me to go down the route without steroids I think because…it does have a really high success rate. I won't deny that. (Iris, female, 20 years old, eczema for 16 years, SKINS project)

Two participants described how they had experienced side-effects (red, heated, and stinging skin) from using a topical calcineurin inhibitor.

Compared to TCS, young people expressed fewer concerns regarding the safety of emollients. A few participants explained how emollients can *"block"* their skin and one expressed concerns about the flammability of emollients containing paraffin.

Participants found that topical treatments, specifically emollients that are applied regularly in large amounts, can be unpleasant in texture and/or smell, can make it difficult to use certain object (such as a pen), can rub off onto other items, such as your clothes or bedding, can make you feel hot, sweaty or *"dirty"*, and can sting when applied. Some avoided applying creams in public as they did not want others to *"find out"* they had eczema. They worried that others could smell their treatments, or that people would not want to touch them (e.g. partners holding their hand) if they had creams on. However, some explained how such worries reduced as they got older and started to care less about the others' opinions.

For peer review only - http://bmjopen.bmj.com/site/about/guidelines.xhtml

> *My colleagues often see me moisturising at my desk. They don't mention it [laughs] And if people think it's a bit weird, like I feel bad, but also like it's so uncomfortable when you can't moisturise.* (Willow, female, 23 years old, eczema since birth, SKINS project)

Burden of eczema self-management

Participants explained how burdensome eczema self-management could be. Applying topical treatments, identifying and avoiding irritants/triggers (especially as there are potentially so many), resisting the urge to scratch, and the 'trial and error' process of finding effective and acceptable (e.g. does not sting) treatments were described to be time-consuming, effortful, frustrating, and sometimes expensive.

> *It was quite a repetitive experience of just back and forth and back and forth. You'd explain [to the doctor], "oh okay well it's not working", like "okay let's, let's higher the dosage. Or let's try and find some steroids in", it just would not work.* (Raashid, male, 24 years, eczema since birth, SKINS project)

> *Some days you just can't be bothered. If you're out with friends, you know it's time to put on your cream; you would ignore it and during that night, I'd sometimes suffer the consequences…and I was scratching and itching all the time.* (Qamar, male, 18 years old, eczema for 11 years, SKINS project)

As can be seen in the above quote, treatment burdens led some young people to skip treatments, especially when they had little time or energy.

Overcoming barriers to eczema self-management

Young people listed several practical barriers to eczema self-management and shared strategies they found helpful for overcoming these. Participants found it challenging to use their topical treatments (mainly emollients) when outside of the home. Specifically, it was

For peer review only - http://bmjopen.bmj.com/site/about/guidelines.xhtml

difficult to use creams during class or take them when travelling abroad on a plane (due to luggage restrictions); or apply them if they worked in a public-facing job (e.g. retail or waiter). Other barriers to accessing treatments included running out of treatments, being unable to get a doctor's appointment to request more treatment (especially if they are living away from home and need to register with a GP), or treatment brands becoming discontinued. One younger participant from the ECO project reported that he found it hard to know how much emollient to apply. Participants found it helpful to keep sample-sized emollients or emollients decanted into smaller containers with them (e.g. in their bag) or around the house, to apply emollient before and after they leave the house, to stock up on treatments to avoid running out, and to be able to buy treatments without a prescription.

Young people explained how it could be difficult to remember to apply topical treatments and emphasised the importance of having a routine for applying treatments (especially their emollients), applying their creams at the same time each day, or making time to apply the creams: *"The biggest thing for fighting eczema is routine…do something that you think is working and do it regularly…don't be disorganised with it."* (Zaahira, female, 21 years old, eczema since birth, SKINS project).

Although young people acknowledged the importance of avoiding irritants and triggers, they explained that it could be difficult to control your environment, for example, staying at a friend's house, or in shared accommodation or an office environment. There were also external factors that they perceived to be unavoidable or outside of their control, such as the weather or stress.

Many participants commented on the cost of topical treatments, and cosmetics or washing products (e.g. shower gels, laundry detergents) that do not cause flare-ups. When they were younger, participants' treatments were free on the NHS, but many had to pay for their

For peer review only - http://bmjopen.bmj.com/site/about/guidelines.xhtml

treatments from the age of 18. This cost prevented some from getting more treatment or led them to buy non-prescription treatments instead (e.g. over the counter medications). Participants particularly resented the cost when buying treatments or products that were ineffective or made their eczema worse.

> *I don't want to go to the doctors and I don't want to get more prescriptions because it just costs so much…once I had to fork out sort of about £36 worth of medications just for one particularly bad flare-up…and then if it doesn't work you're like, 'What, I spent £40 on nothing'.* (Dua, female, 21 years old, eczema for 20-21 years, SKINS project)

Some participants explained how they were still eligible for free prescriptions or obtained prescription prepayment certificates, which helped with their prescription costs.

Taking control of your eczema self-management

Young people shared their experiences of taking control of their own eczema management. They explained how, when they were a child, their parents did the majority of their eczema management. As they became a teenager, they started taking greater responsibility for, and playing a more active role in, their eczema care. Generally, this transition was welcomed by participants and seen as a positive step towards becoming independent, a goal that was important to them.

> *As a child, I wasn't managing [eczema] at all, I'd let my parents do it [applying cream] whenever they felt like it needed being doing. But I wouldn't be applying my cream…And as teen, I realised that it was my problem and that I should be doing it and obviously as a teen you want your independence. You don't want your parents like stripping you off and covering you in bandages and cream.* (Jamila, female, 20 years old, eczema since birth, SKINS project)

For peer review only - http://bmjopen.bmj.com/site/about/guidelines.xhtml

> *My mum has always wanted to come with me to my appointments when I was older. But I've always said, "I want to do it on my own because I want to speak to my doctor." Cos [laughs] she had a habit when I was younger of coming with me to the doctor and…deciding what was being done for me.* (Iris, female, 20 years old, eczema for 16 years, SKINS project)

Like Jamila in the quote above, young people's drive for independence was prompted by a realisation that eczema management was their *"problem"* (rather than their parents' or health professionals') and they would have to live with the consequences of poorly managed eczema. For some, independence was important for maintaining privacy and avoiding their parents seeing them as vulnerable or distressed.

In contrast, some young people highlighted initial apprehension or challenges with these new roles and responsibilities, including moving to an adult clinic, having to do more things for yourself, and taking an active role in their medical consultations.

> *I'm not really looking forward to that because...they don't tell you what to do apparently in the adult clinic...you have to be by yourself in the adult clinic so you have to…pay more attention and they're not going to help me out as much as the children's nurse would.* (Adi, male, 17 years old, eczema for 6.5-7 years, SKINS project)

Although young people were keen to take an active role, some found it intimidating to talk to health professionals or question their suggested treatments.

> *When I go to the doctors I feel like they're the person in charge so I can't really sort of be like, you know, "I know that this suits me best"…I think you do get intimidated…they sort of just give you like, you know that doctor tone, they're just a*

For peer review only - http://bmjopen.bmj.com/site/about/guidelines.xhtml

little bit better than you…and you're like, 'OK I guess you guys are right but [pause]'. (Dua, female, 21 years old, eczema for 20-21 years, SKINS project)

Many participants experienced times when their doctors or pharmacists were reluctant to prescribe their preferred treatments or refer them to a dermatologist. Conversely, some participants shared positive experiences of obtaining their preferred treatments from their health professionals and praised them for listening to them and their willingness to try different treatments. Willow (female, 23 years old, eczema since birth, SKINS project) explained how she asked her GP if she could be given a topical calcineurin inhibitor after a friend with eczema had recommended it. Although her GP prescribed it for her, she felt frustrated that she had to find out about this herself and the GP had not recommended it to her earlier. Young people stressed the importance of doing your own research around treatments (e.g. looking online), being *"confident"* or *"insistent"* when communicating your needs and preferences to health professionals, and writing a list of things to discuss beforehand.

Despite their changing role, the young people's families were still an important source of support for them: driving them to appointments, providing advice on eczema management, helping them apply topical treatments in difficult to reach areas, helping them understand what the doctor said, and speaking on their behalf at medical appointments when they felt unable to. This support was particularly important for early-mid adolescents whose parents were still involved in their eczema management.

[When I apply treatments depends on] how I'm feeling at the time. So if Mum's at the point of, 'Oh [participant name], go put your creams on', I'm like, 'Okay, I'll go put my creams on' and I'll do it. Then I'll come downstairs and she's double checking, 'Oh have you done your creams?' At times she'd say it and I'll say, 'Yes, I'll go do it and then I'll

For peer review only - http://bmjopen.bmj.com/site/about/guidelines.xhtml

completely forget,' and that [is] pretty much the days where I would not do them. (Ben, male, 14 years old, eczema for 6 months, ECO project)

When deciding whether to engage in specific self-management behaviours, participants considered whether doing so was worth the potential impact it would inevitably have on their eczema. For example, decisions around avoiding irritants/triggers were influenced by how difficult it was to avoid (e.g. if it was their favourite food, if everyone around them is drinking alcohol or eating a certain food), their belief about how much it would make their eczema worse, and whether any action could be taken to minimise these consequences (e.g. by applying emollients or taking antihistamines afterwards).

If I cut out lactose I don't think that it would make that much of a difference, so I might as well just eat the cheese and be happy. (Eevi, female, 22 years old, eczema since birth, SKINS project)

I'd probably just carry on doing them [sports] and just sort of live with the consequences so, as long as you…go and have a shower afterwards and…cool down…if I'm having fun doing something then I'm not going to stop just cos of eczema. (Lanie, female, 24 years old, eczema for 20+ years, SKINS project)

Participants were aware of the negative consequences of scratching, however, many found it difficult to avoid scratching completely. This was because a lot of their scratching was done sub-consciously, it was a habit, many of the self-management strategies (e.g. tapping) were ineffective, or the relief of scratching was rewarding.

I'm terrible for scratching. I cannot stop myself. It's like an addiction cos it relieves such a horrible sensation all round my body. (Iris, female, 20 years old, eczema for 16 years, SKINS project).

For peer review only - http://bmjopen.bmj.com/site/about/guidelines.xhtml

I normally try to stop, like stop myself from doing it, but it gets to the point where it's just all over, all over my neck and I just have to scratch it. (Amelia, female, 15 years old, eczema for 12 years, ECO project)

DISCUSSION

Our findings provided a more in-depth understanding of young people's unique experiences of eczema self-management. Specifically, the challenges they face when learning how to take an active role in their self-management: an issue that has not been explored in previous research. Although the new roles and responsibilities around eczema management were generally welcomed by young people, they also brought initial apprehension and challenges. These findings mirrored previous research highlighting that young people often feel unprepared when transitioning from paediatric to adult care.[17,18]

Participants in this study found it hard to communicate their needs and treatment concerns to health professionals, which meant that, at times, they left consultations feeling like they were not listened to or were dissatisfied with the recommended course of action. Our previous qualitative study with this group found that young people with eczema have a strong need to feel understood and 'taken seriously' by their health professionals.[12] The more confident and competent young people feel in communicating their needs to health professionals, the more understood and validated they are likely to feel. There is a need for health professionals to acknowledge and encourage young people's desire to become active contributors to their own health care, treating them as an equal partner in their care, while recognising that this new role may be initially daunting for some.[19]

Our study explored how young people decide whether to engage in behaviours that would exacerbate their eczema (e.g. irritants/triggers, scratching). Previous research has focussed predominantly on the perceived benefits and costs of topical treatments.[10] Young people's

For peer review only - http://bmjopen.bmj.com/site/about/guidelines.xhtml

decisions were influenced by their beliefs regarding the negative consequences of the behaviours, and their perceived control over the behaviour and its negative consequences. People with eczema have a desire to live a 'normal' life and not allow their eczema management impact on their everyday lives.[10,12] Behaviour change interventions can help promote self-management and, as argued by the self-determination theory, such interventions are more effective if they enhance an individual's need for autonomy and minimise conscious effort and lifestyle disruption.[20–22] It is important to support young people to decide which of these behaviours can be avoided and how to minimise the consequences of unavoidable irritants/triggers, focusing on what is positive and possible.[19]

The Burden of Treatment Theory[23] provides a useful framework for interpreting our study findings and making implications for practice. It argues that health interventions can minimise treatment burdens by building an individual's cognitive and material capacity to perform self-management tasks. In the context of our findings, this may involve building a young person's knowledge and skills required for making informed treatment decisions and weighing up the benefits and costs of self-management behaviours. For example, the financial cost of treatments is a common barrier amongst young people.[10] To build their material capacity, health professionals should acknowledge young people's concerns around the costs and educate them regarding any financial support that is available to them. The Burden of Treatment Theory argues that interventions should build and strengthen 'relational networks' (i.e. social networks, health professionals) around patients, and equip them to effectively navigate healthcare systems. Eczema interventions should build young people's confidence and communication skills and support them to make decisions, so they can direct their own care and feel prepared when transitioning to adult clinics.[19]

There are several limitations to this study. Secondary qualitative analysis has been criticised for not considering the socio-cultural-political context under which the primary research was

For peer review only - http://bmjopen.bmj.com/site/about/guidelines.xhtml

conducted.[24] This issue is particularly salient for this study as the two sets of primary data were collected at two different time points. However, to minimise such decontextualisation, avoid any misinterpreted data, and ensure the final interpretations were representative of both data sets, the interviewers of both data sets were co-authors (DG, AMcN).

Although both primary studies explored the topics relevant to the aims of the current study, these studies were conducted for different purposes (i.e. to inform two different health interventions), which may have introduced differences in the data. For example, sections of the transcriptions and, with participant consent, audio and video clips from the SKINS interviews were to be published online. Therefore, compared to the ECO study participants, SKINS participants may have been more motivated and more likely to give socially desirable answers. Despite this, our analysis highlighted that there was a great deal of convergence between the two data sets and our use of disconfirming case analysis ensured that any differences were identified and acknowledged. Our findings are also congruent with other datasets.[9,10,17,18]

As this was a secondary data analysis, it was not possible to carry out additional interviews in order to have full confidence that data saturation was reached. Moreover, the sample included only eight males. Additional interviews with adolescents aged 13-16 and males with eczema additional may have enabled us to explore any differences between the age groups and between genders in more depth. In conclusion, our findings highlight key self-management challenges faced by young people with eczema. Behaviour change interventions must address the treatment concerns of this group and equip them with the knowledge, skills and confidence to take an active role in their eczema management.

For peer review only - http://bmjopen.bmj.com/site/about/guidelines.xhtml

ACKNOWLEDGEMENTS

We are grateful to the Medical Sociology and Health Experiences Research Group (University of Oxford) and Healthtalk.org for data sharing, and the young people who took part in this research. This manuscript presents independent research funded by the National Institute for Health Research (NIHR). The views expressed are those of the authors and not necessarily those of the NHS, the NIHR or the Department of Health and Social Care.

COMPETING INTERESTS

The authors have no competing interests to declare.

FUNDING

This study was funded by the National Institute for Health Research (NIHR) Programme Grants for Applied Research (RP-PG-0216-20007). Data collection for the SKINS project was funded by NIHR under its Research for Patient Benefit scheme (PB-PG-0213-30006).

CONTRIBUTOR STATEMENT

KG, DG, IM, AR, AMcN, SL and MS were involved in designing and planning the study. DG and AMcN were responsible for recruitment and data collection. KG and DG led on the data analysis, with support from MS and IM. AR (PPI representative) and AMcN reviewed and provided feedback on the analysis and interpretations of the study findings. KG drafted the manuscript, with support from DG, MS, and IM. MS, DG, IM, AR, AMcN, and SL critically reviewed the manuscript, contributing important intellectual content and approved the final manuscript.

DATA SHARING STATEMENT

The data that support the findings of this study are available from the corresponding author upon reasonable request.

For peer review only - http://bmjopen.bmj.com/site/about/guidelines.xhtml

REFERENCES

1 Ring J, Zink A, Arents BWM, *et al.* Atopic eczema: Burden of disease and individual suffering - results from a large EU study in adults. *J Eur Acad Dermatology Venereol* 2019;33:1331–40. doi:10.1111/jdv.15634

2 Maksimović N, Janković S, Marinković J, *et al.* Health-related quality of life in patients with atopic dermatitis. *J Dermatol* 2012;39:42–7. doi:10.1111/j.1346-8138.2011.01295.x

3 Peters AS, Kellberger J, Vogelberg C, *et al.* Prediction of the incidence, recurrence, and persistence of atopic dermatitis in adolescence: A prospective cohort study. *J Allergy Clin Immunol* 2010;126:590-595.e1-3. doi:10.1016/j.jaci.2010.06.020

4 Illi S, Von Mutius E, Lau S, *et al.* The natural course of atopic dermatitis from birth to age 7 years and the association with asthma. *J Allergy Clin Immunol* 2004;113:925–31. doi:10.1016/j.jaci.2004.01.778

5 Margolis JS, Abuabara K, Bilker W, *et al.* Persistence of mild to moderate atopic dermatitis. *JAMA Dermatology* 2014;150:593–600. doi:10.1001/jamadermatol.2013.10271

6 National Institute for Health and Care Excellence (NICE). Atopic eczema in under 12s: diagnosis and management (Clinical guideline 57). 2007. https://www.nice.org.uk/guidance/cg57 (accessed 27 Nov 2020).

7 Sav A, King MA, Whitty JA, *et al.* Burden of treatment for chronic illness: A concept analysis and review of the literature. *Heal Expect* 2015;18:312–24. doi:10.1111/hex.12046

8 Eton D, Ramalho de Oliveira D, Egginton J, *et al.* Building a measurement framework

For peer review only

of burden of treatment in complex patients with chronic conditions: A qualitative study. *Patient Relat Outcome Meas* 2012;3:39–49. doi:10.2147/prom.s34681

9 Kosse RC, Bouvy ML, Daanen M, *et al.* Adolescents' perspectives on atopic dermatitis treatment-experiences, preferences, and beliefs. *JAMA Dermatology* 2018;154:824–7. doi:10.1001/jamadermatol.2018.1096

10 Teasdale E, Muller I, Sivyer K, *et al.* Views and experiences of managing eczema: systematic review and thematic synthesis of qualitative studies. *Br J Dermatol* Published Online First: 12 June 2020. doi:10.1111/bjd.19299

11 Ghio D, Muller I, Greenwell K, *et al.* 'It's like the bad guy in a movie who just doesn't die': A qualitative exploration of young people's adaptation to eczema and implications for self-care. *Br J Dermatol* 2020;182:112–8. doi:10.1111/bjd.18046

12 Ghio D, Greenwell K, Muller I, *et al.* Psychosocial needs of adolescents and young adults with eczema: A secondary analysis of qualitative data to inform a behaviour change intervention. *Br J Health Psychol* Published Online First: 26 August 2020. doi:10.1111/bjhp.12467

13 McNiven A. 'Disease, illness, affliction? Don't know': Ambivalence and ambiguity in the narratives of young people about having acne. *Health (Irvine Calif)* 2019;23:273–88. doi:10.1177/1363459318762035

14 Braun V, Clarke V. Using thematic analysis in psychology. *Qual Res Psychol* 2006;3:77–101. doi:10.1191/1478088706qp063oa

15 Malterud K, Siersma VD, Guassora AD. Sample size in qualitative interview studies: Guided by information power. *Qual Health Res* 2016;26:1753–60. doi:10.1177/1049732315617444

For peer review only

For peer review only - http://bmjopen.bmj.com/site/about/guidelines.xhtml

16 Hennink MM, Kaiser BN, Marconi VC. Code Saturation Versus Meaning Saturation: How Many Interviews Are Enough? *Qual Health Res* 2017;27:591–608. doi:10.1177/1049732316665344

17 Fegran L, Hall EOC, Uhrenfeldt L, *et al.* Adolescents' and young adults' transition experiences when transferring from paediatric to adult care: A qualitative metasynthesis. *Int J Nurs Stud* 2014;51:123–35. doi:10.1016/j.ijnurstu.2013.02.001

18 Betz CL, Lobo ML, Nehring WM, *et al.* Voices not heard: A systematic review of adolescents' and emerging adults' perspectives of health care transition. *Nurs Outlook* 2013;61:311–36. doi:10.1016/j.outlook.2013.01.008

19 National Institute for Health and Care Excellence (NICE). Transition from children's to adults' services for young people using health or social care services. 2016. https://www.nice.org.uk/guidance/ng43/resources/transition-from-childrens-to-adults-services-for-young-people-using-health-or-social-care-services-pdf-1837451149765 (accessed 27 Nov 2020).

20 Deci EL, Ryan RM. The 'what' and 'why' of goal pursuits: Human needs and the self-determination of behavior. *Psychol Inq* 2000;11:227–68. doi:10.1207/S15327965PLI1104

21 Ryan RM, Deci EL. Self-determination theory and the facilitation of intrinsic motivation, social development, and well-being. *Am Psychol* 2000;55:68–78. doi:10.1037/0003-066X.55.1.68

22 Yardley L, Morrison L, Bradbury K, *et al.* The person-based approach to intervention development: Application to digital health-related behavior change interventions. *J Med Internet Res* 2015;17:e30. doi:10.2196/jmir.4055

For peer review only

For peer review only - http://bmjopen.bmj.com/site/about/guidelines.xhtml

23 May CR, Eton DT, Boehmer K, *et al.* Rethinking the patient: Using Burden of Treatment Theory to understand the changing dynamics of illness. *BMC Heal Serv Res Serv Res* 2014;14:281. doi:10.1186/1472-6963-14-281

24 Ruggiano N, Perry TE. Conducting secondary analysis of qualitative data: Should we, can we, and how? *Qual Soc Work* 2019;18:81–97. doi:10.1177/1473325017700701

FIGURE LEGENDS

Figure 1 Visual representation of themes and key findings

For peer review only

For peer review only - http://bmjopen.bmj.com/site/about/guidelines.xhtml

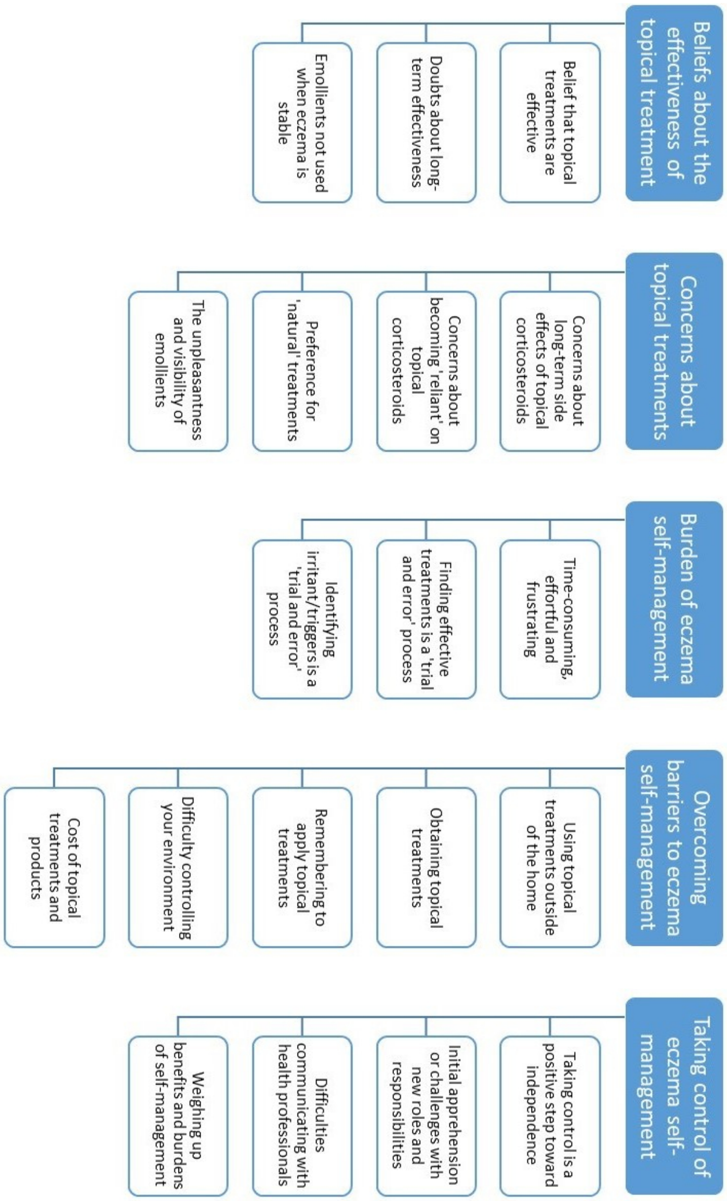

Visual representation of themes and key findings

Supplementary File 1 - Interview Schedule for SKINS Project

Part 1 of the interview: open ended/free-flow

- Could you tell me about your experiences of having [skin condition(s)]. You can say as a little or as much as you like about this.

Part 2 of the interview: follow-up and prompts

Realising that you had skin condition(s)

- What it was like when you first noticed that you might have a skin-related condition (or multiple skin-related conditions)?
- How long after you noticing/having symptoms did you talk to someone else about it?
- Have you been to see a doctor about your [skin condition(s)]? If so, at what stage did you first go?

Early knowledge/information about skin condition(s)

- What was it like when you were told you had [skin condition(s)]?
- Do you recall what, if anything, you knew about [skin condition(s)] before your diagnosis?
- After being diagnosed, did you look for other/more information elsewhere? If so, could you tell me about it?

Medical encounters/meetings

- Could I ask a bit more about the healthcare you've received for your [skin condition(s)]? So things like who do you see, where, how often?
- *If relevant:* What has it been like to move from a paediatric clinic to teenager and/or onto an adult clinic?

For peer review only - http://bmjopen.bmj.com/site/about/guidelines.xhtml

- Could you tell me about what happens when you go see [various health professionals]?

Treatments and managements of skin condition(s)

- Could you tell me about the treatments you have had in the past and any that you now do please?
- What were these treatments like? What did they involve?
- Did you have to make any decisions about these treatments? Like choosing between different options?
- Does anyone help you do these treatments? Or provide instructions about what you needed to do? Do they provide other kinds of practical help around your treatment?
- Are there some treatments or things to manage your [skin condition(s)] which are easier to do than others?
- Are there any which are particularly difficult or upsetting to do?
- Have there been any physical side effects to the treatments for your [skin condition(s)]?
- Have the treatments had any impact on the kinds of activities you can or would like to do?
- Have the treatments you've used had any other changes or prompted concerns for you?

Changes to the skin condition over time and in the anticipated future

- Could you run me through a typical day/week/month from the point of view of managing [skin condition(s)]?
- Have there been any changes over the time you've had [skin condition(s)] as to what the condition has been like (better, worse)? Either in relation to treatments or unexpectedly.

For peer review only - http://bmjopen.bmj.com/site/about/guidelines.xhtml

- Are there any more treatments planned or possible options for management in the future for you?

Everyday life as a young person with [skin condition(s)]

- Could you tell me what it is like being a young person with [skin condition(s)] (as opposed to a child or older adult with this)?
- *If relevant:* Do you think that [skin condition(s)] has an impact for you in terms of school/ college/university?
- *If relevant:* How about any impact in relation to employers (full-time, part-time)? This could be present, past and future/prospective employers.
- Are there any ways in which [skin condition(s)] plays a part in your friendships? This might include friendships at school/college/uni and/or at work but also beyond (including online)?
- Could I ask about any impacts of [skin condition(s)] on (potential and/or actual) romantic relationships?
- In your experience, does having [skin condition(s)] make a difference with regards to social situations or events you might attend? In a negative way or in a positive way?
- Are there any particular places you might go for which are affected in any way by [skin condition(s)]? For example, any places/spaces in particular that you would avoid? Or places where you feel more comfortable? Places you've gone to because of [skin condition(s)]?

Home life and [skin condition(s)]

- Are there any other aspects of your home life and lifestyle that you think having [skin condition(s)] affects? If so, how and why?
- What about any impact on your family [parents, siblings etc.] and/or those you live with [e.g. if in independent accommodation]?

For peer review only - http://bmjopen.bmj.com/site/about/guidelines.xhtml

'Body work' and body-image

- Does your [skin condition(s)] affect the things you can wear or otherwise put on your body/skin? For example cosmetics, make-up, clothes, piercings, tattoos, hair dying, depilation.

Subsequent information and support

- Could you tell me about any practical and/or emotional support you've found or received, either from individuals, organisations or websites?
- Are you aware of any support groups either for your condition or more generally for young people?

Sharing information/messages to others

- What would be your advice to other young people with [skin condition(s)]? For example, is there anything from your experience that you think they might be interested to know or might find helpful?
- Is there anything you wish you had known, or anything you still don't know and would like to, about [skin condition(s)]? Are there things you'd like to know about others experiences?
- Based on your experience of [skin condition(s)], is there anything you think doctors and nurses should know? What would be your message to them?

Closing

- Is there anything else about your experience of [skin condition(s)] that I haven't covered but that you think might be important? Or would you like to add to something you've said previously?

For peer review only

Supplementary File 2 - Interview Schedule for Eczema Care Online Project

Introduction

We are trying to find out as much as possible about your experiences of eczema, how you look after your eczema and how you learnt about this, or anything else you wish you knew. There are **no right or wrong answers** to any of these questions – it is just about trying to find out as much as possible in your own words. Any questions before we start?

Eczema views and experiences/perceived causes

- Could you tell me a bit about what its like to have eczema?
    - How does it affect you at the moment?
- What do you think causes eczema/flare ups?

Views and experiences of eczema treatments/self-care

- What kind of things do you do to help your eczema?
    - What treatments do you use for your eczema?
    - What do you think of your treatments?
- Can you tell me what you find easy/difficult about [insert specific]?
    - Could you talk me through maybe a daily or weekly routine of when you apply/use [insert treatment]?
- Could you tell me more about how you use [insert treatment they use]?
    - At home, at school?
    - What is easy/difficult about this?

For peer review only - http://bmjopen.bmj.com/site/about/guidelines.xhtml

- If self-managing, how are you finding looking after your eczema? Or how would you feel about looking after your eczema?

- Do you do anything else to help make your eczema better?
    - Can you tell me what you find easy/difficult about that?
    - Are there also things that you've bought in shops or over the counter treatments that you've tried?
    - How do you try to reduce scratching?
    - How do you try to manage triggers/irritants?
    - Have you ever had anything like bath oils or soap substitutes?
    - Have you ever used relaxation techniques?

Experiences of eczema information provision

- What kinds of information have you had on eczema? From where?
- What's it like talking to health professionals (i.e. GPs, nurses, pharmacists)

Views about Quality of Life

- How does eczema make you feel?
    - Do you have strategies? – tell me more.
- Does eczema have much of an impact at all on what you wear or fabrics that you might choose?
- What about other things that you might use on your body (for example deodorant or perfume)?

Views about websites/web-based interventions

- How would you feel about using a website for information and support on eczema?

For peer review only - http://bmjopen.bmj.com/site/about/guidelines.xhtml

- What sort of things would you like to see on the website?
- What do you think would be helpful or would have been helpful to you in the past?
- We may include videos about eczema. What do you think would be good/not good about videos on eczema?

That is all really useful, thank you. Is there anything that we haven’t talked about that you would like to add?

For peer review only

For peer review only - http://bmjopen.bmj.com/site/about/guidelines.xhtml

Greenwell et al. Taking charge of eczema self-management: a qualitative interview study with young people with eczema

COREQ checklist

No	Item	Guide questions/description	Comments	Location in manuscript
**Domain 1: Research team and reflexivity**				
Personal Characteristics				
1.	Interviewer/facilitator	Which author/s conducted the interview or focus group?	AMcN, DG	Page 5&6
2.	Credentials	What were the researcher's credentials? *E.g. PhD, MD*	KG – PhD, CPsychol.<br>AMcN – PhD<br>DG – PhD, CPsychol	Page 5&6
3.	Occupation	What was their occupation at the time of the study?	KG – Health Psychologist & Senior Research Fellow<br>AMcN – Qualitative Health Researcher<br>DG – Research Fellow and Charted Psychologist	-
4.	Gender	Was the researcher male or female?	All female	-
5.	Experience and training	What experience or training did the researcher have?	All experienced qualitative postdoctoral researchers.	Page 5-6
Relationship with participants				
6.	Relationship established	Was a relationship established prior to study commencement?	Most participants were not known to the interviewers prior to recruitment, but two participants in the SKINS project were.	-
7.	Participant knowledge of the interviewer	What did the participants know about the researcher? e.g. *personal goals, reasons for doing the research*	SKINS - Participants were told that sections of the transcriptions and, with participant consent, audio and video clips from the interviews were to be published online.<br>ECO – Participants were told that the interviews were to help develop a self-management website for young people.	Page 5,21-22
8.	Interviewer characteristics	What characteristics were reported about the interviewer/facilitator? e.g. *Bias,*	Interviewers and data coders was carrying out the research to inform health interventions, which	-

Greenwell et al. Taking charge of eczema self-management: a qualitative interview study with young people with eczema

No	Item	Guide questions/description	Comments	Location in manuscript
		assumptions, reasons and interests in the research topic	could be a potential source of bias. One of the interviewees had visible hand eczema during the interviews, which may have influenced how participants share their eczema experiences.	
**Domain 2: study design**				
Theoretical framework				
9.	Methodological orientation and Theory	What methodological orientation was stated to underpin the study? *e.g. grounded theory, discourse analysis, ethnography, phenomenology, content analysis*	Thematic analysis.	Page 9
Participant selection				
10.	Sampling	How were participants selected? *e.g. purposive, convenience, consecutive, snowball*	Maximum variation sampling/purposive sampling.	Page 5&6
11.	Method of approach	How were participants approached? e.*g. face-to-face, telephone, mail, email*	Mail, face-to-face, telephone, email, research advertisements via social media, patient representative groups, and NHS sites (e.g. posters in waiting rooms, packs handed out by health professionals).	Page 5&6
12.	Sample size	How many participants were in the study?	28	Page 6
13.	Non-participation	How many people refused to participate or dropped out? Reasons?	ECO – Of the 1123 young people approached, 55 agreed to take part in the interviews, 12 were 13-16 years old. Seven of these did not take part in the interview either could not be contacted by telephone or were not selected for purposive sampling.	Page 5&6

For peer review only - http://bmjopen.bmj.com/site/about/guidelines.xhtml

Greenwell et al. Taking charge of eczema self-management: a qualitative interview study with young people with eczema

No	Item	Guide questions/description	Comments	Location in manuscript
			SKINS – One interview participant did not provide consent for their data to be used.	
Setting				
14.	Setting of data collection	Where was the data collected? e.*g. home, clinic, workplace*	At the participants' homes or alternative meeting places.	Page 5&6
15.	Presence of non-participants	Was anyone else present besides the participants and researchers?	Relatives were occasionally present during the interviews.	Page 5&6
16.	Description of sample	What are the important characteristics of the sample? *e.g. demographic data, date*	Demographic information can be found in Table 1. Interviews were carried out during October 2014 and December 2015, and March-May 2018.	Table 1; page 5 & 6
Data collection				
17.	Interview guide	Were questions, prompts, guides provided by the authors? Was it pilot tested?	The interview schedules can be found in Appendix 1 and 2. The SKINS interview schedule was reviewed by PPI representatives.	Appendix 1&2.
18.	Repeat interviews	Were repeat interviews carried out? If yes, how many?	Only single interviews were carried out.	-
19.	Audio/visual recording	Did the research use audio or visual recording to collect the data?	Audio or video (SKINS only) recording.	Page 5&6
20.	Field notes	Were field notes made during and/or after the interview or focus group?	Field notes were made after all interviews.	-
21.	Duration	What was the duration of the interviews or focus group?	SKINS interviews lasted up to 2 hours; ECO interviews between 22-50 minutes	Page 5&6
22.	Data saturation	Was data saturation discussed?	Yes	Page 9-10, 22
23.	Transcripts returned	Were transcripts returned to participants for comment and/or correction?	SKINS participants were sent a copy of the transcripts and they could remove sections and correct or add details if they wished.	Page 6

For peer review only

Greenwell et al. Taking charge of eczema self-management: a qualitative interview study with young people with eczema

No	Item	Guide questions/description	Comments	Location in manuscript
**Domain 3: analysis and findings**				
Data analysis				
24.	Number of data coders	How many data coders coded the data?	Two (KG & DG)	Page 9
25.	Description of the coding tree	Did authors provide a description of the coding tree?	No	-
26.	Derivation of themes	Were themes identified in advance or derived from the data?	Derived from the data (inductive analysis).	Page 9
27.	Software	What software, if applicable, was used to manage the data?	QSR's NVivo 12 Pro was used.	Page 9
28.	Participant checking	Did participants provide feedback on the findings?	SKINS participants were sent a copy of the transcripts and they could remove sections and correct or add details if they wished. PPI provided feedback on summaries of the findings.	Page 7-8
Reporting				
29.	Quotations presented	Were participant quotations presented to illustrate the themes / findings? Was each quotation identified? e.g. *participant number*	Participant quotations are presented and each quotation is identified by a pseudonym and their gender, age and eczema duration is noted.	Page 10-19
30.	Data and findings consistent	Was there consistency between the data presented and the findings?	Yes	Page 10-19
31.	Clarity of major themes	Were major themes clearly presented in the findings?	Yes	Page 10-19; Figure 1
32.	Clarity of minor themes	Is there a description of diverse cases or discussion of minor themes?	Diverse cases are discussed.	Page 10-19.

For peer review only
